# Critical Knowledge Gaps for Shellfish Allergies: Insights from Global Market Presence and Trade of Shellfish

**DOI:** 10.3390/foods15101720

**Published:** 2026-05-13

**Authors:** Dragana Stanic-Vucinic, Mirjana Radomirovic, Xuli Wu, Marija Stojadinovic, Tanja Cirkovic Velickovic

**Affiliations:** 1 Center of Excellence for Molecular Food Sciences, University of Belgrade—Faculty of Chemistry, Studentski trg 16, 11000 Belgrade, Serbia; dstanic@chem.bg.ac.rs (D.S.-V.); mstojadinovic@chem.bg.ac.rs (M.S.); 2 Department of Chemistry, Institute for Chemistry, Technology and Metallurgy, National Institute of the Republic of Serbia, University of Belgrade, 11000 Belgrade, Serbia; mirjana.radomirovic@ihtm.bg.ac.rs; 3 School of Public Health, Health Science Center, Shenzhen University, Shenzhen 518060, China; wxl@szu.edu.cn; 4 Department of Chemistry and Biology, Serbian Academy of Sciences and Arts, Knez Mihajlova 35, 11000 Belgrade, Serbia

**Keywords:** molluscan allergens, crustacean allergens, tropomyosin allergens, shellfish allergen diagnostics, shellfish consumption, shellfish production, shellfish allergen labelling, shellfish allergen detection, food allergies

## Abstract

With the increasing popularity of seafood in human diets, managing allergic reactions to shellfish has become more critical. The objective of this review is a comprehensive analysis of critical knowledge gaps for shellfish allergies based on the relationship of shellfish allergens to global shellfish production and market presence. The methodological approach included the integration of Food and Agriculture Organization (FAO) production data and allergen databases, as well as official legal documents on allergen labeling. According to FAO data, global Mollusca production exceeds that of Crustacea. Despite this, progress in molecular allergen characterization and the development of diagnostic and analytical tools for Mollusca remains underdeveloped. Additionally, food allergen labelling regulations for shellfish are inconsistently applied across countries. Key allergens have been identified in several shellfish species, particularly Crustaceans, but more allergens must be discovered to enhance diagnostic tools. Within Mollusca, Cephalopoda remains understudied, with only one allergen identified despite dominating the shellfish trade. The lack of molecular studies on molluscan allergens hinders the further development of diagnostic tools and accurate allergen detection. Given the high consumption rates and the prevalence of molluscan allergies, large populations are at risk. Therefore, systematic research on molluscan allergens is essential for improving diagnostics, food safety regulations, and public health measures worldwide. Our review summarizes the knowledge gaps of the economically most relevant species of shellfish based on their market presence and trade and provides guidance for further research in the area.

## 1. Introduction

Blue foods, i.e., aquatic foods (fish, invertebrates, algae, and plants) cultured or captured in freshwater and marine environments, including shellfish, are a key source of nutrition worldwide [1], making them 17% of the animal protein supply (Figure S1A) and reaching over 50% in several countries in Asia and Africa [2]. Growing interest has been put on blue foods in terms of current and future transitions to more sustainable food systems since the way in which blue foods are produced has been considered more environmentally sustainable than terrestrial animal-sourced foods [3]. Considering all these factors, in a high-production scenario, driven by increased financial investment and innovation in aquaculture production, the annual global production of aquatic animal-source food is predicted to increase by 8% in 2030 compared to the baseline scenario in which moderate growth in aquaculture production is assumed [4].

Fisheries and aquaculture, according to the Food and Agriculture Organization of the United Nations (FAO), comprise food groups: marine and freshwater fishes, Crustaceas, mollusks (excluding Cephalopodes), and Cephalopodes, with fishes contributing to around 70% of the total aquatic animals produced globally (Figure S1B).

Crustaceas are invertebrates in possession of an exoskeleton and a segmented body of the phylum Arthropoda, class Malacostraca. Crustacea shellfish relevant to the human diet are shrimps/prawns, crayfish, lobster, and crabs. Mollusks are any soft-bodied invertebrate of the phylum Mollusca, usually wholly or partly enclosed in a calcium carbonate shell secreted by a soft mantle covering the body, represented classes of Bivalvia, Gastropoda, and Cephalopoda. The molluscan shellfish relevant to the human diet are gastropods (including abalones, limpets, land and marine snails, and whelks), bivalves (including oysters, mussels, and scallops), and cephalopods (including squids and octopuses) [5]. Both Crustaceas and mollusks are reported to cause allergic reactions. In food allergy studies, shellfish allergens primarily come from two major groups, crustaceans and mollusks.

Increased awareness of the high nutritional value of crustaceans and mollusks has led to a rise in shellfish consumption, and this has been associated with more frequent reports of allergic reactions [6]. In contrast to allergens such as egg and cow’s milk, shellfish allergy does not in general resolve with age, and thus, lifelong dietary avoidance is necessary [7]. Seafood allergy is regarded to persist for life in up to 90% of patients [8]. On the other hand, adults can also develop new food allergies, and certain food allergies, such as shellfish allergies, are more likely to develop during adulthood than others [9].

Shellfish seems to be the most common trigger of immunoglobulin E (IgE)-mediated food allergy among adults in the United States [10]. The prevalence of shellfish allergies is estimated to be up to 3% of the adult population in the United States based on self-report survey studies [11,12]. In Europe, prevalence assessed via questionnaire-based methods was 0.1% to 5.5% for crustacean allergy, 0.4% to 1.5% for mollusk, and 0.1% to 1.5% for shellfish allergy, while food challenges confirmed a 0% to 0.9% prevalence of crustacean allergy and 0.1% prevalence of mollusk allergy [13].

Shellfish allergies have one of the highest rates of severe reaction to ingested food, known as food-induced anaphylaxis. Data from the European Anaphylaxis Registry demonstrated that shellfish and wheat were identified as major triggers of food-induced anaphylaxis in adults, where shrimp was the main food trigger [14]. In adults and children, shellfish-induced anaphylaxis has a prevalence of 0.41% and 0.11% in Asia, 1.9% and 0.72% in North America, and 0.27% and 0.55% in Europe [15]. A recent study showed that 8.3% of European patients with asthma or atopic diseases aged 1–18 years experienced anaphylaxis after shrimp ingestion [16] with prawn and crab (crustaceans) being the major causes of anaphylaxis in both children and adults. In Singapore, Thailand, and Hong Kong, a majority of the food-induced anaphylactic cases were attributed to shellfish, at frequencies of 24.6% [17], 45.2% [18], and 71.2% [19], respectively.

The epidemiology of shellfish allergies varies across countries. Based on a cross-sectional food allergy prevalence survey, in the United States, the prevalence of shellfish allergies in children was 1.3%, with more children allergic to crustaceans (1.2%) than mollusks (0.5%) [20]. Combined crustacean and mollusks allergy self-reportedly affects as many as 5.5% of French children (5–17 years old) and 9.0% of American adults [13]. The prevalence of crustacean allergy alone is considerably higher, with the highest reported prevalence of 10.3% determined among Italian adults who were confirmed positive via a Skin Prick Test (SPT) [21]. A self-report survey study in USA adults reported a 2.4% crustacean allergy prevalence [12], as well as 1.9% for shrimp, 1.3% for lobster, and 1.3% for crab [11].

The prevalence of shellfish allergy is generally higher among Asians, based on questionnaire-based surveys: in China, 15.3%, 13.9%, and 11.1% and 7.3% for crab, shrimp, and shellfish [22], 7.3% in Taiwanese children and adults [23], in Hong Kong, 1.3% for preschool [24] and 1.7% for children aged below 15 years [25], 5.2% in Singapure, and 5.1% in Philippines teenagers [26].

Although numerous clinical cases of sensitized patients to mollusks are often reported, there are still no reliable clinical diagnostic approaches for isolated mollusk allergy due to undefined allergens and extensive immunological cross-reactivity with other allergen sources containing similar proteins in different species [27,28]. In addition, the wide variety of mollusk species available in different regions of the world and cross-reactivity within mollusk species make a diagnosis of mollusk allergy even more complicated [29]. Mollusk allergy was reported in several studies across Europe and Asia [30,31], but its prevalence seems to be lower when confirmed using available in vitro and in vivo tests [5]. Based on a self-report survey study in USA adults, molluscan allergy prevalence was 1.6% [11,12]. Approximately 45% of individuals with a crustacean allergy are also allergic to mollusks, while 70–80% of mollusk-allergic patients also experienced allergic reactions to crustaceans [12,32]. The patients with crustacean or mollusk allergy should avoid all shellfish species not only due to cross-reactivity, but also due to contamination of each other. In addition, patients with allergy to shrimp are at risk of developing clinical allergy to edible insects [33], which are frequently consumed in Asia and, since recently, also in Europe and North America.

Molluscan shellfish allergies have been documented to all classes of mollusks, including gastropods (e.g., limpet, abalone), bivalves (e.g., clams, oysters, mussels), and cephalopods (e.g., squid, octopus) [34]. Of 45 Spanish patients with mollusk allergy, 55% were allergic to cephalopods (squid 40%, cuttlefish 11%, octopus 11%), 31% to bivalves (clam 18%, mussel 9%, razor fish 7%, cockle 2%), and 24% to gastropods (limpet 15%, snail 9%), demonstrating heterogeneity in allergy to mollusks. Although not known with certainty, the prevalence of molluscan shellfish allergy is likely to parallel consumption patterns being more frequent in locales where consumption is frequent [34]. Therefore, although awareness of mollusk allergies is growing, its prevalence still remains uncertain [35].

In addition, Asian countries have distinct ways of seafood processing and dietary habits, introducing seafood early into the infants’ diet, seafood is often consumed raw, and some seafood species that are not being consumed in other parts of the world are consumed [36]. Previous studies of coastal Asian populations reported high rates of shellfish allergy, suggesting a relationship between greater shellfish allergen exposure and shellfish allergy risk. Shellfish allergy was very common in the local Singapore (4–6 years, 1.19%; 14–16 years, 5.23%) and Philippines (14–16 years, 5.12%) [26]. According to a meta-analysis, including studies with questionnaire-based surveys, in China, the prevalence of shellfish allergy was observed in 19% of the total patients with food allergy [37]. Interestingly, in Asia, patients with shellfish and fish allergies are more common than those with peanuts, nuts, and wheat allergies [38].

Thus, several studies suggest that shellfish is the dominant food allergen among Asian populations [26,39]. Shellfish allergy is characterized by the pan-allergenic molecule tropomyosin, discovered in numerous species of Crustacea and Mollusca and described as the abundant, thermostable, muscle protein tropomyosin (TPM), which shows high structural homology between phylogenetically related species [5,40,41]. Interestingly, although over 80% of European shellfish-allergic subjects were sensitized to tropomyosin (TPM) as the main shellfish allergen, a recent study suggested that only half of Asian subjects with challenge-proven shrimp allergy were sensitized to TPM [36]. The low sensitization rate to TPM was also reported in Singapore (34.2%) [42] and Japan (37%) [43]. Similarly, Chinese patients were shown to be sensitized to multiple shrimp allergens, supporting the opinion that TPM might not be the major allergen in Asians [36]. Diversified allergen sensitization in Asians is explained by the consumption practice for shrimp and crab non-muscle body parts (cephalothorax, containing brain, heart, stomach and bladder, ovaries, and hepatopancreas) together with muscles [36].

Despite the large number of edible shellfish, only 62 shellfish allergens, of which 52 are from crustaceans and 10 from mollusks, have been identified, were characterized and registered in the WHO/IUIS Allergen nomenclature database by October 2024. Knowing that there are thousands of species in the human diet, of which there are more than 800 species of only Mollusca Bivalvia in the human diet [44], prioritization is a must.

This review aims to provide a comprehensive overview of shellfish production, the regulatory landscape, allergens, and the diagnostic and analytical repertoire of available tools and provides insight into the most relevant gaps to be filled to improve shellfish allergy diagnosis, management, and public health measures worldwide.

## 2. Methodology

Production data was retrieved from FAO FishStat, FAO global fishery and aquaculture statistics, for 2021 (https://www.fao.org/fishery/en/fishstat, accessed on 13 December 2023), using FAO’s Fisheries and Aquaculture statistics software FishStatJ (https://www.fao.org/fishery/en/statistics/software/fishstatj, version 4.03.00, accessed on 13 December 2023). For each species, there are one or several entries for each producing country, as production data are separately provided for different FAO major fishing areas (such as Northern regions, central regions, and Southern regions), as well as for the type of production (such as capture production and aquaculture production). In order get information on the total production of shellfish live weight for a given species by a given country, if there was more than one entry, the tons produced in different FAO major fishing areas and different types of production were summed. Total production of shellfish live weight for a given species by a given country was calculated as the % of total production of shellfish live weight for a given species by all countries. The official WHO/IUIS Allergen Nomenclature Database (https://allergen.org/) was used for the review of shellfish allergens registered by WHO/IUIS. Allergens listed in WHO/IUIS were retrieved in October 2024. The Allergome data base (https://www.allergome.org/index.php), as of October 2024, was retrieved to collect information on shellfish allergens non-registered by the WHO/IUIS. Publications were additionally reviewed manually until September 2024 to added to the list on non-registered allergens based on reported IgE reactivity. A Blast similarity search was performed with sequence information in the UniProt database (https://www.uniprot.org/uniprotkb, version 2.14.0) available as of November 2024. For the regulatory landscape, official regulative documents of the EU, USA, China, and Balkan countries (Republic of Serbia, Montenegro, and Bosnia and Hercegovina) were used as sources of data. Additional sources of data were official reports of corresponding authorities of the EU and USA. Commercially available shellfish allergens for allergy diagnostics was searched from web sites and catalogues of the most prominent manufacturers from USA, Europe, and China (Phadia/Thermo Fisher Scientific, Siemens Healthineers, Faber, Macroarray Diagnostics, ALK-Abelló, STALLERGENS GREER, HollisterStier Allergy, Beijing Macro-Union Pharmaceutical Limited Corporation, Diagnostische Systeme & Technologien GmbH, and Labor Dr. Weyers). The following databases were consulted for scientific publications: PubMed, PubMed Central (PMC), ScienceDirect, Wiley Online Library, Scopus, and Web of Science.

## 3. Overview of Global Production of Shellfish

### 3.1. Production of Shellfish

**Figure 1:**
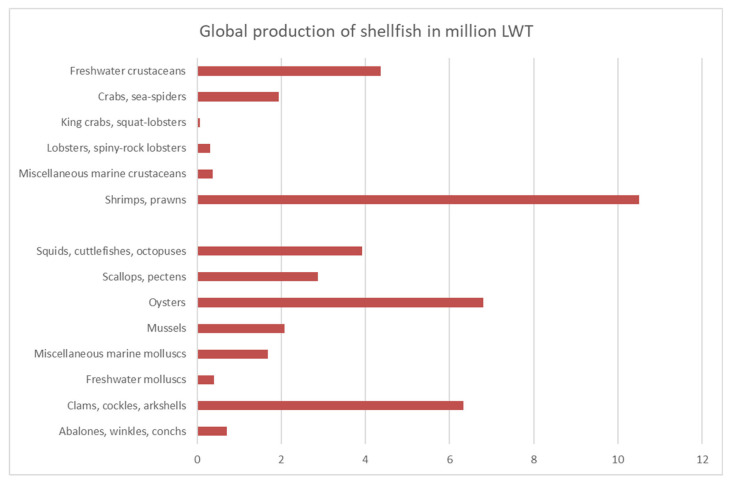
Global production of shellfish in 2021 in million LWT according to FAO database (https://www.fao.org/fishery/en/statistics/software/fishstatj, accessed on 13 December 2023). Production includes both captured and aquacultured shellfish.

Of the 494 taxonomically recognized species ever farmed in the world of aquaculture and fisheries, 88 species are mollusks and 49 species are crustaceans. Crustacean production in 2021 amounts to nearly 18 million tons of live weight (LW) (Figure 1). In comparison, FAO reported the annual production of crustaceans across the world at approximately 16.9 million tons in 2020 [2].

Data for 2021 show that Mollusk and Cephalopodes, with 24,8 million live weight tonnage (LWT, the total weight of fish at the time of capture, before any processing) produced globally, account for nearly 60% of the global production of shellfish foods (Figure 1). FAO FishStat gives separate production data for Cephalopodes: they contribute to around 10% of the global production of all shellfish foods. Among the produced Mollusk, on top are oysters and clams (Supplementary Information, Table S1).

**Figure 2:**
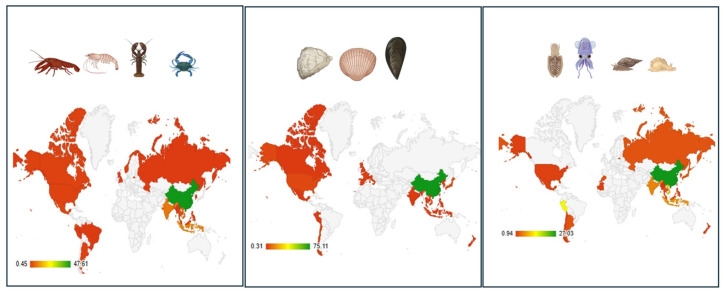
Global shellfish production in 2021. Geochart of top 20 countries with the highest Crustaceas production in 2021 (**left panel**). Geochart of top 20 countries with the highest Mollusca (ex. Cephalopoda) production in 2021 (**middle panel**). Geochart of top 20 countries with the highest Cephalopoda production in 2021 (**right panel**). The data are mined from FAO. Created in BioRender. Velickovic, T. (2025) https://BioRender.com/r99t719.

China mainland was the main producer of Crustaceas, Mollusca, and Cephalopoda in 2021 (Figure 2, Supplementary Tables S2–S4). Marine shrimps dominate the global production of crustaceans, and they are an important source of foreign exchange earnings for a number of developing countries in Asia and Latin America (Supplementary Table S5). Supplementary Table S5 gives an overview of the most important crustacean shellfish in 2021, including aquaculture and capture production. The species produced in >1000 tones and their main producers are presented. The top produced marine crustacean species is whiteleg shrimp (*Penaues vannamei/Litopenaeus vannamei*), while red swamp crawfish is the top products among freshwater crustaceans. Among molluscan shellfish, various cupped oysters and Japanese carpet shell (*Ruditapes philippinarum*) are the main products (Table S6).

Jumbo flying squid (*Dosidicus gigas*) is a top Cephalopode produced globally, amounting to nearly 1 million LWT annually, just from the top two producers. Cephalopod production is dominated by squids, quite opposite to the production of mollusks, where various oysters, mussels, clams, and scallops comprise the top 10 produced foods (Supplementary Table S6).

**Figure 3:**
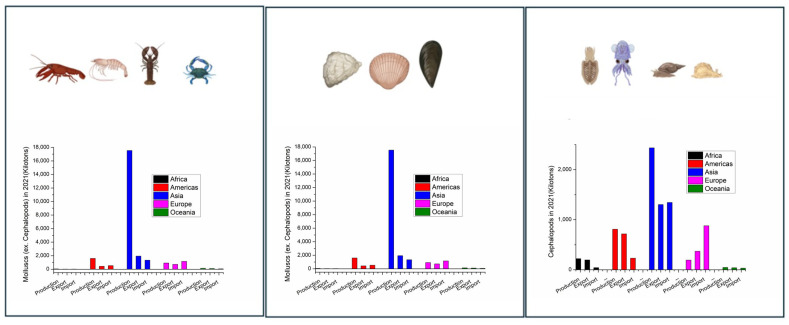
Overview of shellfish trade. Production, export, and import of crustaceans (**left panel**), mollusks (excluding Cephalopods) (**middle panel**), and Cepahalopods (**right panel**) in different regions of the world for 2021. The data are mined from the FAO database using FishStat.

Of the shellfish produced globally, the global trade of crustaceans dominates the market (Figure 3). Mollusca (ex. Cephalopodes), though making the top produced group, accounts for the minor fraction of global trade of exported commodities. Export comprises live, frozen, chilled, dried, cured, and meat of shellfish etc. (Supplementary Tables S7–S12).

Mollusca makes 48% of all shellfish being globally produced, but only 7% is exported (with a similar % of imported commodities based on Mollusca), and most of the produced Mollusca are in fact consumed in the countries producing them. China produces over 80% of the world’s bivalves, but domestically consumes almost all of this production (Supplementary Tables S7–S12).

Mollusca, a heterogeneous group of species, grouped according to FAO into Mollusca ex. Cephalopodes and Cephalopodes (such as squids) rational, has quite a different trading pattern. Mollusca ex. Cephalopods are mostly consumed in the producing country, while Cephalopodes trade accounts for nearly 64% of the production, with import equally dominating the shellfish market. Thus, the production of Mollusca (ex Cephalopods) could, with a 5% margin of error, be related to the consumption in the producing country (oysters, clams, mussels).

The European market is characterized by the highest import of all commodities based on Mollusca and Crustaceas ([45].

### 3.2. Shellfish Supply per Capita

**Figure 4:**
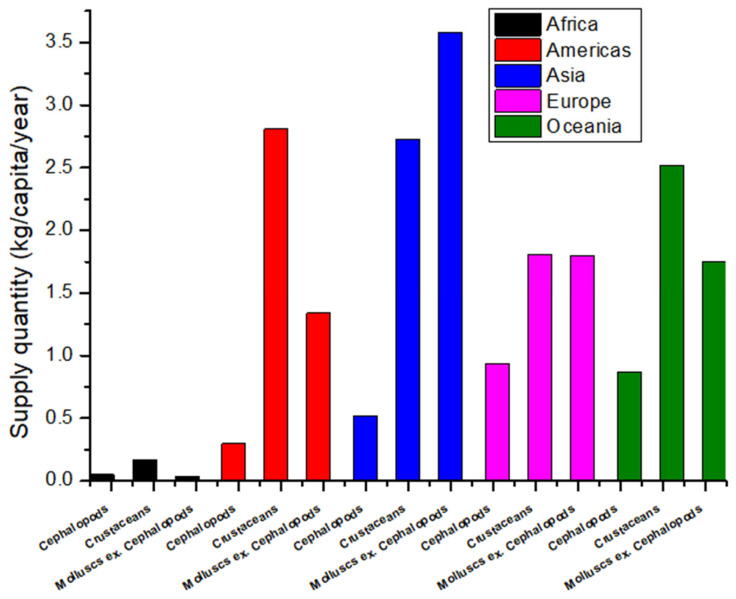
Supply quantity (kg/capita/year) of Cepahalopods, crustaceans, and mollusks (excluding Cephalopods) per region in 2021.

According to 2021 FAO data, although Asia is the highest producer, importer, and exporter of all shellfish groups (Figure 3), its supply quantity per capita, e.g., availability for consumption, is relatively low, particularly Cephalopodes (Figure 4 and Section S2.2).

Although Asia is by far the highest producer, importer, and exporter of all shellfish groups (Figure 3), only several Asian countries were in the top 20 countries with the highest shellfish supply quantity per capita in 2021 (Table S13). China and its SARs/Provinces and the Republic of Korea have the highest shellfish supply quantity per capita, suggesting in general the highest consumption of shellfish per capita. Although China mainland is the highest producer (Table S3), exporter (Table S7), and importer (Table S10) of Cephalopoda, its supply quantity, and thus consumption per capita per year, is not in the top 20 countries for Cephalopoda (Table S13).

### 3.3. Consumption of Shellfish

Although there are available consumption data for fish and seafood as a whole, data on shellfish consumption is still scarce, particularly for different shellfish species and for different regions/countries. Another issue is the regular updating of shellfish consumption data, which rapidly changes due to changes in eating habits, as well as shellfish availability. Shellfish consumption data are of great importance not only for food allergy risk assessment, but also for exposure and risk assessments of different environmental contaminants found in shellfish.

Available data from 2019 show that of the 20.5 kg of seafood per capita consumption, nearly 75 percent came from finfish and the remainder came from shellfish, of which 12% were mollusks (excluding cephalopods), 11% crustaceans, and 2% cephalopods [2]. Countries with the highest seafood consumption include coastal nations such as Iceland or the Maldives, at more than 80 kg per capita per year on average, while landlocked countries such as Afghanistan, Ethiopia, and Tajikistan consume less than 1 kg per year.

According to the European Market Observatory for Fisheries and Aquaculture Products, the EU is a major world market for fishery and aquaculture products: in 2021, apparent consumption amounted to 10.60 million tons in live weight equivalent (LWE), corresponding to 24 kg LWE per capita. Consumption, however, varies greatly across the EU, from 56 kg LWE per capita in Portugal to 6 kg LWE per capita in Hungary. Portugal still stands out as the major EU consumer of fishery and aquaculture products. The main products consumed are tuna (mostly canned), salmon, cod, Alaska pollock, shrimps, mussel, hake, and herring (https://www.eumofa.eu/the-eu-market#euFishMarket, accessed on 17 September 2024). Of that, the consumption of shellfish species per capita in Europe was as follows: shrimps 1.47 kg, mussel 1.23 kg, squid 0.62 [46]. For the US, according to data from the National Oceanic and Atmospheric Administration (NOAA) in 2020, the average American consumption of fresh and frozen seafood products was 14.6 pounds, of which shellfish accounted for a total of 6.3 pounds per capita [47]. From the US National Fisheries Institute’s (NFI) recently released top 10 list of seafood consumption in 2021 (https://aboutseafood.com/about/top-ten-list-for-seafood-consumption/, accessed on 17 September 2024), of total seafood consumption per capita per year (20.50 pounds) in 2021, the consumption of the top 10 seafood was 15.67 pounds, of which 6.75 pounds consumed are shellfish (0.26 clams, 0.59 crab, and 5.9 shrimp).

Relative to production, Asian countries, particularly China mainland, have low shellfish consumption per capita per year (Table S13). Moreover, although China mainland is the highest producer of Cephalopoda, its supply quantity, and thus consumption per capita per year, is not in the top 20 countries, e.g., its consumption is only 0.56 kg/capita/yr. On the other hand, small costal countries, although globally negligible producers of shellfish, have high consumption per capita per year. Similarly, relative to production, European countries are also very high consumers of shellfish.

## 4. Regulatory Landscape

The best management of food allergic diseases is the avoidance of food containing causative allergens. With raw agricultural materials, avoiding allergens is simple. However, in processed foods, allergens can be hidden or added unintentionally to food products as a processing residue or a contaminant. Therefore, processed food should be labeled for intentionally added allergens. In addition, food businesses may voluntarily provide information about the unintentional presence of allergens to fulfil legal obligations to provide safe food. This precautionary allergen labeling (PAL) is usually in the form “may contain x” or “not suitable for consumers with a x allergy”. Unintentionally added allergens may be present in food products due to allergen cross-contamination, when there is a risk that an allergen can enter the product accidentally during the production process, manufacturing, handling, transport, or storage of foods. Legislation in the EU and USA enforces regulations requiring companies to list ingredients on foods and beverages prepacked for direct sale, as well as food sold in loose form in, i.e., restaurants, food from a takeaway, and foods sold loose in retail outlets. For the labeling of allergens in the EU, the name of the allergen or allergenic ingredient must be clearly highlighted from the rest of the text, e.g., with different letters, styles, or colors in the background. In USA, the allergen’s food source name must be declared at least once on the food label in one of two ways, in parentheses following the name of the ingredient in the ingredient list or immediately after or next to the list of ingredients in a “contains” statement. For certain foods or substances that cause allergies or other hypersensitivity reactions, there are more specific labeling requirements. In the USA, legislation requires a listing for Big 9 causative allergen sources and, in EU, for 14 sources of allergens.

The analytical detection of allergens from crustaceans is part of USA regulations on food allergen labeling. The FDA enforces regulations requiring companies to list ingredients on packaged foods and beverages. For certain foods or substances that cause allergies or other hypersensitivity reactions, there are more specific labeling requirements. The Food Allergen Labelling and Consumer Protection Act of 2004 (FALCPA) identified eight foods as major food allergens: milk, eggs, fish, crustacean shellfish, tree nuts, peanuts, wheat, and soybeans. On 23 April 2021, the Food Allergy Safety, Treatment, Education, and Research (FASTER) Act was signed into law, declaring sesame as the 9th major food allergen recognized by the United States. The change was effective on 1 January 2023. However, although under section 201(qq) of the Federal Food, Drug, And Cosmetic Act (As Amended Through P.L. 118–15, Enacted 30 September 2023), crustacean shellfish (such as crab, lobster, or shrimp) and ingredients that contain protein derived from crustacean shellfish are regarded as major food allergens; molluscan shellfish (such as oysters, clams, mussels, or scallops) are not [48].

The EU lists 14 allergenic sources that must be declared according to the legislation of the European Union (Regulation (EU) N° 1169/2011 of the European Parliament and of the Council 2014) [49], including fish, crustaceans, and mollusks and products thereof.

In China, there is no national legal regulation regarding food allergen labeling. In 2008, the Beijing Municipal Government issued the Local Standard (DB11/Z5-21-2008) “Olympic Food Safety—Food Allergen Labelling” [50]. Food safety for Beijing Olympic Games food allergens labeling DB11/Z521-2008), which was the earliest regulation on food allergen labeling in China, was abolished after the Olympics. In April 2011, the National Standards Committee of China issued the National Standard (GB7718-2011) “National food safety standard-General rules for the labelling of prepackaged foods” [51]. According to this standard, for any food or its products contain the eight major categories of “allergic foods” defined by FAO/WHO, it is advisable to use an easily recognizable name in the ingredient list or to indicate it in a nearby location. But this is recommended labeling.

Within the Food Allergy Research and Resource Program (FARRP), the Institute of Agriculture and Natural Resources of University of Nebraska (updated in August 2024, https://farrp.unl.edu/IRChart, accessed on 7 October 2024) provided information on international food allergen labeling regulations. This database with 42 entries includes countries, unions (EU, 27 EU countries and 6 non-EU countries adopting EU allergen labeling regulations), GSO (6 countries), Central America (5 countries), and CARICOM (21 member states), e.g., in total, 105 countries and their regulatory requirements. Most African countries do not have regulations on the labeling of food allergens. There are big differences in food allergen legislation among non-EU European countries. Some non-EU European Countries adopted EU allergen labeling regulations: Iceland, Liechtenstein, Norway, Macedonia, Switzerland, United Kingdom (UK).

Out of 42 entries listed, all regulate crustacean shellfish, except Israel (38 all Crustacea, Japan and South Korea only shrimp and crab, Thailand only crab and shrimp), but only 13 entries, e.g., 52 countries, regulate Mollusca shellfish (11 entries, e.g., 44 countries, regulate all Mollusca, 7 GSO countries only clams, and South Korea only abalone, mussel, oyster, and squid). Although molluscan allergen labeling is not obligatory, in Japan, there is recommended labeling for abalone and squid, while in Thailand, recommended is labeling for cuttlefish, neritic squid, octopus, escargot, mussel, clam, oyster, scallop, mytilus, meretrix lusoria, and abalone. Countries not included in the overview of the University of Nebraska, such as non-EU European Countries of the West Balkan, all regulate fish, crustaceans, and Mollusca (Montenegro [52], Serbia [53]), except Bosnia, which does not regulate Mollusca [54].

Thus, of the top ten most populated countries (India, China, USA, Indonesia, Pakistan, Nigeria, Brazil, Bangladesh, Russian Federation, and Ethiopia), only in the Russian Federation is molluscan shellfish allergen labeling regulated (there is no data for Pakistan and Ethiopia). This means that in these countries, which are contributing to 50% of the world population, molluscan shellfish allergen labeling is not regulated.

Each country regulates a list of the most relevant food allergens for labeling, based on epidemiological screening and dietary habits of its population. Proper legislation and the control of food products for allergen labeling, as well as unintentionally added food allergens (as contaminants) by state regulatory agencies, are a major step towards the better management of food allergic diseases that may prevent unwanted severe allergic reactions, especially for the vulnerable pediatric population.

These data show that most countries still do not regulate molluscan shellfish allergen labeling, presenting a health risk for the large portion of the world population. With increasing rates of molluscan shellfish allergies worldwide, the recognition of molluscan allergens by policy makers in these countries is urgently needed to ensure that people living with food allergies can safely purchase and consume foods. This is particularly important for people from countries with molluscan shellfish allergen labeling legislation who travel to countries without it. However, more and more countries include molluscan shellfish as allergens in their updated regulatives.

## 5. Allergens of Shellfish

The two invertebrate groups of crustaceans and molluscan are, for culinary reasons, often combined as shellfish but belong to two very different phyla. The evolutionary and taxonomic diversity of the various consumed seafood species poses a challenge in the identification and characterization of the major and minor allergens critical for reliable diagnostics and therapeutic treatments. Allergens in various crustacean and mollusk species are mainly found in the edible portions of meat. Several crustacean allergens have been identified over the past two decades, due to the advances in protein identification and allergen-characterization techniques.

### 5.1. Shellfish Allergens Registered in WHO/IUIS

The WHO/IUIS registered 57 Arthropoda (e.g., 52 crustacean allergens and 5 allergens of Bombyx mori (Silk moth)) and 10 molluscan (including one allergen of *Helix aspersa* [*Cornu aspersum*] (Brown garden snail) food allergens (Tables S14 and S15). A comprehensive review on shellfish allergens in 2018 [5] reported 31 crustacean and 6 mollusk allergens, suggesting that progress has been made mostly with regards to new crustacean allergens. Currently, of 61 shellfish molecules registered as allergens by the WHO/IUIS Allergen Nomenclature Sub-Committee, only 9 belong to mollusks. Taking into account for example, that the molluscan major allergen tropomyosin (TPM), a highly homologues protein across species, shares only up to 60% amino acid sequence identity with the crustacean TPM [55], identification and registration of more mollusk shellfish allergens are urgently needed.

**Figure 5:**
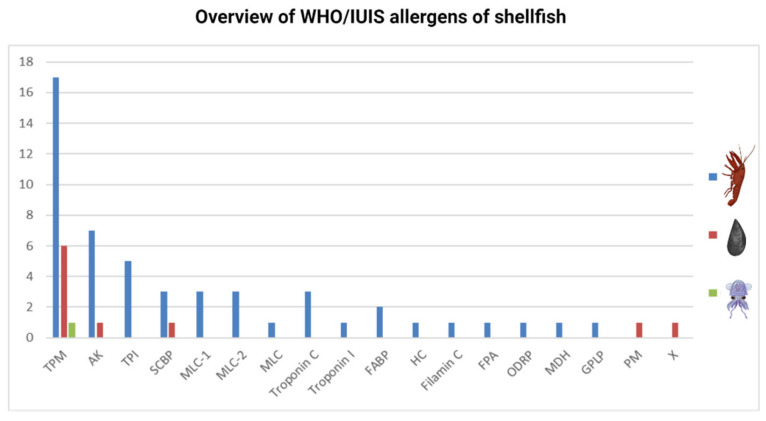
Overview of WHO/IUIS-registered allergens of shellfish. Allergic proteins registered in Crustacea and Mollusca are grouped according to protein type. TPM—tropomyosin, PM paramyosin, AK –arginine kinase, X -unknown, SCBP—sarcoplasmic calcium-binding protein, HC—hemocyanin, PPH—phosphopyruvate hydratase, PK—pyruvate kinase, MLC—myosin light chain, MDH—malate dehydrogenase, FPA—fructose 1,6-bisphosphate aldolase, ODRP—ovary development related protein, GPLP—glycophosprylase like protein, FABP—fatty acid binding proteins. Blue (Crustace), red (Mollusca ex Cephalopodes), and green (Cephalopodes). Created in BioRender. Velickovic, T. (2025) https://BioRender.com/b38o566.

Crustacean allergens are proteins found mostly in muscle tissue, the cephalothorax, or in roe. Their physiological functions are involved in muscle movement and energy metabolism. Several allergens have also been identified from the edible parts of mollusk species. Three allergenic proteins, tropomyosin, arginine kinase, and triosephosphate isomerase, have also been identified in both shellfish groups (Figure 5).

Of the identified food allergens from the phylum Arthropoda, sub-phyllum Crustacea, all originate from blue food, e.g., from class Malacostraca and order Decapoda, including freshwater and marine shrimps, prawns, crabs, lobsters, and crayfishes. Of the identified food allergens from the phylum Mollusca, except allergens from the brown garden snail *Helix aspersa*, all originate from marine blue food, from the classes Bivalvia (oysters), Gastropoda (abalone and whelk), and Cephalopoda (squid).

Of 52 Crustacea shellfish food allergens and their isoforms (52 allergens and 2 isoforms), the majority are tropomyosins (Figure 5). Of 10 molluscan shellfish food allergens and one isoform, 6 allergens are TPMs. Of all Mollusca, Cephalopods, with only one registered allergen (from Japanese flying squid), represent a significantly understudied allergenic seafood source.

Of the class Bivalvia, there are no registered allergens of clams, cockles, mussels, scallops, and numerous other families that live in saltwater, as well as a few families that live in freshwater.

Only in the period 2019–2021, more than 106 new allergens have been added to the WHO/IUIS [56]. Important discoveries included paramyosin, Rap v 2, an allergenic paramyosin in mollusks. Paramyosins were previously known mainly as arthropod allergens [56]. However, although there are hundreds of commercially important edible shellfish species, in the WHO/IUIS, allergens from only 22 crustacean and from only 8 molluscan species are currently registered.

### 5.2. Shellfish Allergens Not Registered in WHO/IUIS

**Figure 6:**
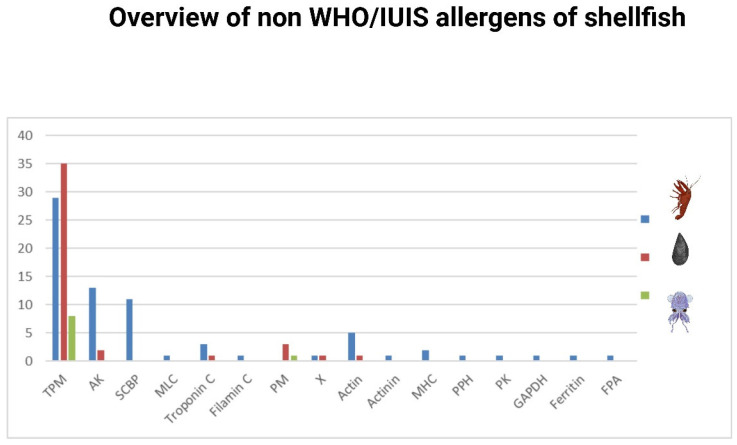
Overview of non-WHO/IUIS registered allergens of shellfish grouped according to protein type. Blue (Crustacea), red (Mollusca ex Cephalopodes), and green (Cephalopodes). TPM—tropomyosin, PM paramyosin, AK—arginine kinase, X—non-available, SCBP—sarcoplasmic calcium-binding protein, MLC—myosin light chain, MHC—myosin heavy chain, HC—hemocyanin, GAPDH—glyceraldehyde hehydrogenase, FPA—fructose 1,6-bisphosphate Aldolase. An overview of all non-registered allergens, their available sequences, and availability as recombinant allergen is are given at https://cherry.chem.bg.ac.rs/handle/123456789/7052, date accessioned 7 April 2025. Created in BioRender. Velickovic, T. (2025) https://BioRender.com/b38o566.

In addition to 52 TPM Crustacea shellfish allergens registered in the WHO/IUIS, 72 allergens from different Crustacea species, which are IgE reactive, were reported (Figure 6), of which, one third are TPMs. This is similar to the registered Crustacea allergens, of which, one third are TPMs. These nonregistered allergens need to be further tested in order to be approved by the WHO/IUIS. Non-registered proteins with IgE reactivity in Mollusca are 52, of which 9 are described in Cephalopoda (Figure 6, in detail, data available at https://cherry.chem.bg.ac.rs/handle/123456789/7052, date accessioned 7 April 2025). In addition to 6 TPM Mollusca allergens registered in the WHO/IUIS, 43 TPMs from different Mollusca species, which are IgE-reactive, were reported. As for registered Mollusca allergens, the vast majority are TPMs, and this suggests that in Mollusca shellfish, the allergen repertoire is poorly characterized.

In contrast to registered shellfish molluscan TPMs encompassing only oyster, abalone, whelk, and squid species, unregistered IgE-reactive allergens were also shown in clam, sea snail, cockle, mussel, and scallop, as well as in Cephalopoda octopus and cuttlefish species. All of these nonregistered TPMs are solid candidates for future approval by the WHO/IUIS, as TPM has shown to be the major and the most dominant molluscan allergen. Besides TPM, there are numerous IgE-reactive shellfish allergens, which are still non-registered in the WHO/IUIS, including paramyosin, arginine kinase, sarcoplasmic calcium-binding protein, myosin light chain, myosin heavy chain, hemocyanin, glyceraldehyde hehydrogenase, fructose 1,6-bisphosphate aldolase, and allergens with unknown function (Figure 6 and Section S3).

### 5.3. Availability of Natural and Recombinant Shellfish Allergens

Based on the WHO/IUIS allergen registry, the most comprehensive allergen profile was delineated from *Penaues monodon*, comprising nine registered shrimp allergens, including TM, AK, MLC2, SCBP, Troponin, HC, TIM, FABP, and GP. These allergens are mostly identified via conventional immunodetection methods. The availability of the genome assembly data of penaeid shrimp was indispensable for studies of penaeid shrimp allergenic proteins [57] as it allowed the production of recombinant molecules and their allergenicity testing. Similarly, with more genome assembly data, particularly from Mollusca, research will make significant progress.

Beside these listed IgE-reactive allergens, not yet registered, there is a high number of shellfish proteins that have been identified in different species and/or in silico predicted as allergens, but IgE reactivity was not demonstrated yet. In addition, there are numerous shellfish species for which potential allergens have to be identified in the future. This implies that the identification of potential allergens in neglected shellfish species and testing of their IgE reactivity is also huge gap in the shellfish allergy field, particularly important for species consumed worldwide and/or consumed by a globally high population, such as Asian. All submitted requests for allergen inclusion in the WHO/IUIS list must fulfil the molecular and immunological requirements for inclusion into the allergen nomenclature database, including IgE-binding data using purified natural or recombinant allergens.

It implies that further testing of these non-registered allergens and their registration are urgently needed in order to widen the list of shellfish allergens approved by the WHO/IUIS. This is particularly important for molluscan allergens, where there is a nearly five times higher number of molluscan IgE-reactive non-registered allergens than registered ones (Figure 6). A necessary prerequisite is the availability of natural or recombinant allergens of shellfish.

#### 5.3.1. Availability of Natural Shellfish Allergens

Unfortunately, purified natural shellfish allergens are currently hardly available. The only commercially available one is shrimp TPM, by InBio (https://inbio.com/, accessed on 7 October 2024). However, not only is it costly, but it also does not originate from a single species. According to the manufacturer, depending of the season, this preparation originates from *L. setiferus*, *F. aztecus*, and *F. duorarum*. Therefore, in order to obtain natural shellfish allergens, research groups have to isolate and purify them. This includes the development of methods for isolation and purification, which is usually multistep and complex, and characterization of the purified protein. In addition, usually very low yields are obtained, with several isoforms present, and preparations may differ from batch to batch. Thus, the isolation and purification of natural shellfish allergens could be very time consuming and costly, as reflected in the commercially poor availability of natural shellfish allergens.

#### 5.3.2. Availability of Recombinant Shellfish Allergens

In contrast to natural allergens, the production of recombinant allergens is cost effective, and preparations contain only one isoform and with no batch-to batch differences. The most shellfish allergens registered in the WHO/IUIS are commercially available as recombinant proteins produced in different expression systems (*E. coli*, yeast, baculovirus-infected insect cells, mammalian cells, and vitro *E. coli*) (Section S3).

In order to be registered in the WHO/IUIS, IgE-binding data must be obtained using sufficiently purified (natural or recombinant) allergens (https://allergen.org/submission.php, accessed on 7 October 2024). Therefore, the commercial availability of a much wider palette of recombinant non-registered shellfish allergens would enable IgE testing of a much higher number of shellfish allergens and their faster registration, particularly molluscan allergens, which are currently scarce (only 10 registered). As obtaining highly purified natural shellfish allergens is complex and costly, the commercial availability of recombinant allergens is a key prerequisite for the more efficient and up-to-date introduction of shellfish allergens in the WHO/IUIS list in the future.

## 6. Diagnostic and Therapy Tools for Shellfish Allergies

According to the recently published European Academy of Allergy and Clinical Immunology guideline, providing recommendations for diagnosing IgE-mediated food allergy, food allergy diagnosis starts with an allergy-focused clinical history followed by tests to determine IgE sensitization: skin prick test (SPT), serum allergen-specific IgE (sIgE) to extracts, sIgE to allergen components, and basophil activation test (BAT). SPT and sIgE to allergen extracts are recommended as first-line tests, and sIgE to individual allergen molecules is the second line. If available, BAT can be undertaken in equivocal cases, should standard allergy tests be insufficient to provide a diagnosis. Oral food challenge (OFC) remains the reference standard and should be reserved for cases that cannot be clarified with SPT, sIgE and/or BAT, while double-blind placebo-controlled food challenge (DBPCFC) can be used if the open OFC is equivocal or for research purposes [58] For in vivo allergy diagnosis, several types of skin tests are used, including the skin prick test (SPT), intradermal test (IDT) and patch test, while in vitro diagnosis includes a total IgE assay, serum specific IgE assays against allergen sources/molecules, and the basophil activation test (BAT) [59]. Besides clinical assessments, the current clinical shellfish allergy diagnosis includes SPT, sIgE testing, and OFC.

Despite OFC being the diagnostic standard for food allergy, there are no standardized protocols for shellfish allergies, as observed in international multicenter cohorts of patients from the United Kingdom and USA [60].

### 6.1. Shellfish Allergens Within Skin Prick Test Diagnostic Tools

There are several issues related to SPT for shellfish allergies. Firstly, commercially available shellfish extracts are limited compared to the wide variety of dietary shellfish. From Table S16, it could be observed that three most reputable manufacturers of allergen extracts (ALK-Abelló, STALLERGENS GREER, and HollisterStier Allergy) offer a very narrow palette of only several shellfish extracts. These manufacturers do not offer extracts of freshwater crustaceans, mussels, squids, octopuses, and abalones. In contrast, Chinese manufacturer Beijing Macro-Union Pharmaceutical Limited Corporation offers only extracts from two species, but for which China is the highest producer (>95%). Some manufacturers, such as HollisterStier Allergy, do not provide the information of shellfish species.

This problem could be partially solved through the in-house preparation of extracts from fresh shellfish for SPT, as done by Asero et al. [61], or via a prick-to-prick test using fresh or cooked food, as done by Scala et al. [62]. However, both approaches lack standardization.

Secondly, even commercially available shellfish allergen extracts for SPT still lack standardization. Several studies demonstrated that commercial shellfish SPT extracts from different manufacturers have different compositions and heterogeneous protein profiles. Moreover, Asero et al. [61] demonstrated that five commercially available crustacean SPT extracts displayed a dramatic loss of protein bands compared to fresh shrimp extract when analyzed via SDS-PAGE. These authors also found a heterogeneous abundance of three shellfish allergens in these commercial extracts, resulting in 32 clinical profiles among 157 shrimp-allergic patients. Ruthers et al. [63] examined 11 commercial crustacean and 5 molluscan SPT extracts, utilizing biochemical and immunological methods and mass spectrometry. They showed not only that the total protein content varied up to 14-fold (0.1–1.4 mg/mL), but also, in the SDS PAGE profiles, 1–15 distinct bands were visible, suggesting huge differences in the antigen repertoire, as well as their contents in extracts, between different manufacturers. Further, the authors realized that some proteins may have been degraded into smaller fragments in some extracts. In addition, utilizing serum from five shellfish-allergic subjects, IgE-binding patterns to 14/16 extracts underlined high variance in anticipated in vitro and in vivo potency and no IgE binding to 2 of the tested extracts, due to low protein and allergen contents, concluding that there is a high risk of false-negative SPT results with these extracts. In addition, a recent study investigated the influence of the geographic location of capture or aquaculture on the allergenic protein profiles of black tiger shrimp (*Penaeus monodon*) and demonstrated that ten of the twelve known shrimp allergens were detected, but with considerable differences between locations, while tropomyosin abundance varied by up to 13 times between locations [60]. This shows that the shrimp origin might directly impact the readout of commercial crustacean allergen detection kits, most of which target tropomyosin. In addition, as in Asia, shrimp is always served head-on and shell-on, for the Asian population, shrimp cephalothorax extracts should be standardized and made available for the clinical diagnosis of seafood allergy [31]. Therefore, the standardization of allergen extracts is urgently needed to improve the accuracy and reliability of SPT. On the other hand, in vitro component-resolved diagnostic tools might be beneficial and enable better predictions regarding cross-sensitization, especially for individuals sensitized to only one shellfish allergen [63].

### 6.2. Shellfish Allergens Within Diagnostic Tools for Measurement of Allergen-Specific IgE

Several manufacturers produce shellfish allergen extracts that can be used for specific IgE, IgG4, and IgG determination, degranulation tests, BAT, cell tests, lymphocyte transformation tests, and kit-manufacturing (such as, DST and Labor Dr. Weyers, Table S17). However, even these manufacturers offer extracts from a limited number of shellfish species, and many species with the highest global production and consumption are neglected. For example, there is no commercially available extract of whiteleg shrimp, whose production and consumption is the highest of all shellfish species.

Shellfish allergen-specific IgE in human sera can be measured in vitro using several ELISA kits, or via using singleplex and multiplex measurement platforms. Most ELISA kits are seafood or shellfish allergy tests, thus measuring total IgE to the mixture of all seafood (fish and shellfish) or shellfish allergens from several species. Although these tests are useful, they cannot measure specific IgE to separate shellfish species.

Component-resolved diagnosis (CRD) that involves the detection of sIgE to individual allergenic molecules and/or allergen peptides has emerged to possibly resolve the ambiguities of extract-based SPT and sIgE analysis. Molecular diagnosis using single allergens, instead of whole food extracts, to quantify sIgE levels was shown to more accurately reflect the clinical reactivity. However, the relevant allergen components fit for diagnosis may, however, vary among different geographical locations depending on the dietary habits and disease prevalence.

There are several platforms that measure shellfish -specific IgE levels against a chosen allergen analyte, which can be a recombinant or purified native allergen component or allergen extract from one or several species (Table S17). Singleplex platforms, such as ImmunoCAP (Phadia/Thermo Fisher Scientific, Uppsala, Sweden), IMMULITE (Siemens Healthcare Diagnostics, Los Angeles, CA, USA), and FABER (Centri Associati di Allergologia Molecolare), measure specific IgE levels against a single chosen allergen analyte. Multiplex platforms, such as Alex2 Allergy (Macroarray Diagnostics, Wien, Austria) and ImmunoCAP ISAC 112 (Thermo Fisher Scientific) use a fixed array of more than one hundred allergen analytes.

In the ImmunoCAP System, 17 shellfish extracts (including 4 different shrimps under single test code f24) are available for routine sIgE quantification (Table S17), including 8 for Crustacea and 9 for Mollusca (4 from Bivalvia, 3 Cephalopoda, and 2 Gastropoda class) (https://www.abacusdx.com/media/PU_Product%20Catalogue%202022.pdf, accessed on 19 August 2024). Unfortunately, currently, there is only one ImmunoCAP with an allergen component, rPen a 1 Tropomyosin from shrimp (*Penaeus aztecus*). IMMULITE 1000/2000/Xpi offers 11 shellfish extracts (4 for Crustacea and 7 for Mollusca) and also only one allergen component, nPen m 1 from Penaeus monodon (shrimp). In FABER there are 6 available shellfish extracts and three allergen components, Lit v 1 from *Litopenaeus vannamei* (shrimp), Ven ga 1 from *Venus gallina* (clam), and Uro du 1 from *Uroteuthis duvauceli* (squid). In multiplex Alex2 Allergy Explorer, of an array of 288 allergens, 10 extracts and 5 allergen components are from shellfish. All allergen components are from shrimps (nPen m 1, nPen m 2, nPen m 3, and rPen m 4 from *Penaeus monodon*, rPen a 1 from *Penaeus aztecus*, and rCra c 6 from *Crangon crangon*). The ImmunoCAP ISAC 112 platform contains 112 allergen components, of which only 3 allergens are from shellfish (nPen m 1, nPen m 2, and nPen m 4 from Penaeus monodon).

This data also shows that different platforms offer shellfish extracts from different species and that shellfish extract palettes in all of these platforms are still very limited. In addition, extracts from many shellfish species with the highest global production and consumption are still missing, particularly species with the highest consumption in Asia (Supplementary Tables S5 and S6). Therefore, singleplex and multiplex platforms should widen shellfish extract palettes, and, for choosing further species for extract production, they should be guided also by monitoring global production and particularly consumption. For example, only FABER offers an extract and one allergen component from *Litopenaeus vannamei* (whiteleg shrimp), the species with the highest global production/consumption among all shellfish.

On the other hand, despite the fact that there are 62 (52 Crustacean and 10 Molluscan) food allergens in the database of WHO/IUIS, only 7 crustacean and 2 molluscan allergens are commercially available as allergen components within singleplex and multiplex diagnostic tools. Therefore, the detection of mollusk hypersensitivity must still rely on molluscan extracts or on an indirect molecular diagnosis based on the presumption of cross-reactivity with crustaceans, unless proven with the oral food challenge. The total amount and repertoire of shellfish consumed have increased worldwide, particularly in the last 10 years, due to the increased availability of shellfish in supermarkets, increased consumption of shellfish because of its nutritional values and health benefits, and increased travel to countries where seafood is a common part of the diet. As a consequence, there is a growing prevalence of shellfish allergies. Therefore, manufacturers of diagnostic tools for shellfish allergies should follow this trend and expand the palette of diagnostic tools in a timely manner. For example, despite the fact that available ImmunoCAP shellfish extracts are very limited compared to the variety of dietary shellfish, this repertoire was not widened by Phadia/Thermo Fisher Scientific in the last 9 years, not even for a single one-shellfish extract (Catalog for 2022 https://www.abacusdx.com/media/PU_Product%20Catalogue%202022.pdf, accessed on 19 August 2024 vs. Catalog for 2013 https://www.abacusdx.com/wp-content/uploads/2015/05/Phadia-Allergy-Autoimmunity-Product-Catalogue.pdf, accessed on 19 August 2024).

For example, ImmunoCAP ISAC, containing only three shrimp allergens (Pen m 1, Pen m 2, and Pen m 4) allowed a diagnosis of only 44% of patients [64], while by using a comprehensive panel of 11 recombinant shrimp allergens, Wai et al. [65] achieved a detection rate of >80%. In addition, although TM is a common sensitizing allergen across populations, the major shrimp allergens could differ among populations [65] Therefore, a much wider repertoire of allergen components for the measurement of allergen-specific IgE is needed for more accurate and reliable shellfish allergy diagnostics, particularly for molluscan shellfish.

Developing countries are the most significant producers and consumers of shellfish. However, although food allergies, in general, are currently a significant health care problem in the developing world, food allergies are under-recognized as a clinical specialty, which compromises the chance for an accurate diagnosis. The main challenges are as follows: lack of reliable data on the prevalence of food allergies, skin testing issues, in vitro test accessibility, settings and personnel for testing, as well as management issues with respect to local circumstances [66].

### 6.3. Quantification of Shellfish Allergens for Food Safety and Monitoring

Analytical methods for the detection and quantification of shellfish allergens have mostly been developed for crustacean tropomyosin and target either the whole protein, peptide fragment, or gene segment encoding tropomyosin. The sensitivity of some methods for the quantification of TPM protein are so high, reaching the limit of detection down to even 20–30 pg/mL [67,68]. Most of the methods for the detection and quantification of shellfish TPM are based on tropomyosin recognition by specific antibodies. The ELISA sandwich assay is the most frequently used method for the detection and quantification of TPM [69,70,71]. Methods based on liquid chromatography-mass spectrometry (LC-MS) use the TPM peptide fragment as the target [72,73,74]. In all mentioned studies, crustacean TPM was the target, and there are only a few studies reporting methods for the quantification of molluscan TPM-specific recognition. There is only one published method based on TPM recognition by specific molluscan TPM antibodies. Reference [75] developed a sandwich ELISA method for the detection of TPM from molluscan bivalves (clam, scallop, and cockle), based on a monoclonal antibody against a C-terminal peptide of TPM, which is conserved across crustacean and molluscan species. The multiplex polymerase chain reaction (PCR) method with capillary electrophoresis for the detection of TPM from oyster, mussel, abalone (gastropod species), and clam was developed by Sathe and Sharma [76]. Similarly, Suh et al. [77] developed a multiplex PCR assay combined with capillary electrophoresis for the simultaneous detection of TPM allergens from oyster, mussel, abalone, and clam mollusk species. Unfortunately, methods developed for the quantification of crustacean TPM cannot be applied for the quantification of molluscan TPM. It has been shown that clam residues were detected quite weakly using a commercial crustacean tropomyosin kit, quantifying only 0.4 μg/g of tissue, in contrast to shrimp where it detected 3389 μg/g of tissue [78]. A detailed presentation of the latest evaluation and detection methods of shellfish allergens could be found in several recent reviews by [28,79,80,81].

Currently, there is no commercially available pure natural molluscan TPM, nor antibodies against it. Commercially available are 5 recombinant TPMs registered in the WHO/IUIS and a ready to ready-to-use ELISA kit for the detection of molluscan TPM based on tropomyosin from *Helix aspersa* (garden snail) TPM.

## 7. Cross-Reactivity of TPM from the Economically Most Relevant Species of Shellfish with WHO-Registered Allergens

It is usually considered that cross-reactivity between two proteins is probable if their sequence identity is >70%, which also implies that the proteins have a similar 3D fold [82]. In such cases, linear sequence alignment methods (i.e., BLAST) are frequently used to predict cross-reactivity between allergenic proteins.

Based on the global production of shellfish (FAO FishStat for 2021) presented in the Supplementary Materials (Tables S5 and S6), the top 5 economically most relevant species or groups of Crustacea and Mollusca grouped into freshwater Crustacea, crabs, lobsters, shrimps/prawns, oysters, mussels, clams, scallops, squids, and freshwater Mollusca have been included in the cross-reactivity search. For the economically most relevant species without any TPM registered (or reported as IgE-binding), information on the sequence availability for TPM was collected. Furthermore, for those TPMs, a BLAST (version 2.14.0) search was performed to identify the homologues proteins among existing allergens. Detailed results of the homology search between the registered TPM of shellfish and corresponding proteins of the most economically relevant species are presented in the supplementary data available online. Only the top homologous allergen is presented in the overview given in Table 1. Sequence identity refers to the percentage of amino acids that show a direct match with the compared sequence during alignment.

From Table 1, it can be concluded that TPMs of the most economically relevant crustacean shellfish have >90% of homology with all crustacean TPM allergens registered in the WHO/IUIS, and half of them have homology >98%. On the other hand, homology with all molluscan TPM allergens registered in the WHO/IUIS is >60%.

Table 2 shows that the most economically relevant molluscan shellfish TPMs for which sequence information exists show >70% of homology with all molluscan TPMs and >50% homology with all crustacean TPM allergens registered in the WHO/IUIS (not considering homologies with only available partial sequences). The highest homologies of the most economically relevant molluscan shellfish TPMs are with Sac g 1 and Hal l 1. The only exception is flat oyster, *Ostreus edulis*, for which partial TPM sequence information exists, showing only 55% homology with TPM of the closely related Pacific cupped oyster *Crasosstrea gigas* (*Magelana gigas*).

### Identification of Critical Knowledge Gaps on Shellfish Allergens

Based on the global production of shellfish (FAO FishStat for 2021) presented in the Supplementary Materials (Tables S5 and S6), the top 5 economically most relevant species or groups of Crustacea and Mollusca have been included in the overview tables (Table 1 and Table 2), grouped into the following: freshwater Crustacea, crabs, lobsters, shrimps/prawns, oysters, mussels, clams, scallops, squids, and freshwater Mollusca. Information presented in the overview for each species/genus include the following: production data and main producers, availability of in vivo and in vitro diagnostic tools, and commercially available individual allergens, regardless of whether natural or recombinant; allergens in the WHO/IUIS (and nonWHO/IUIS). If no information on the allergens was available, the availability of genomic information on TPM sequences was searched in the UniProt database for the species or species representing genera. Finally, BLAST linear sequence similarity search results are shown as described above.

For all entries without the availability of any of the most critical information on allergenicity, diagnostic tool availability, or sequence information, species are marked in red to highlight critical information that is missing.

**Figure 7:**
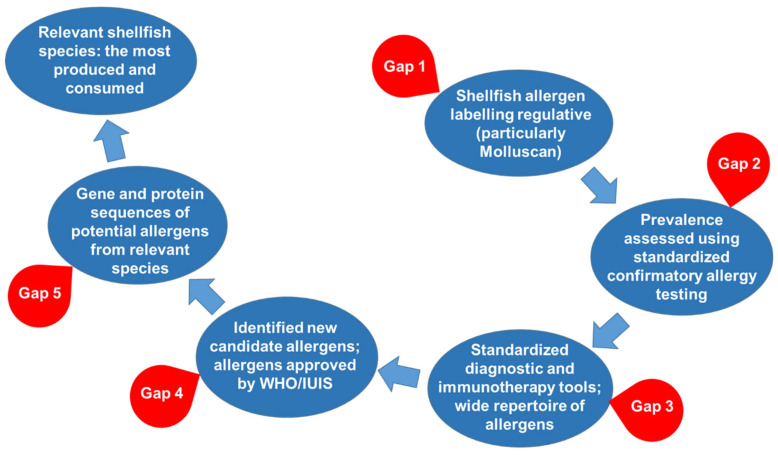
Overview of critical gaps in shellfish allergy and their dependency relationships.

In general, several critical gaps have been identified related to shellfish allergy (Figure 7):

**Shellfish allergen labeling regulative gaps**: Of 195 countries in the world today, only 105 regulate crustacean shellfish allergen labeling, while molluscan shellfish is regulated only by 52 countries. The reason for the absence of molluscan allergen labeling in most countries is probably the fact that molluscan shellfish are not in the Codex Alimentarius priority allergen list [83]) due to the lack of data on the prevalence, severity, and/or potency. Therefore, legislation on shellfish allergen labeling, particularly for molluscan shellfish, is urgently needed, which is of increasing importance due to globalization in terms of trading and traveling. Prerequisites for introducing regulations on shellfish labeling are reliable data on prevalence, severity, and potency, particularly data obtained based on standardized confirmatory allergy testing, as well as warning epidemiology of shellfish-induced anaphylaxis, primarily for molluscan shellfish.

**Prevalence, severity, and potency gaps:** Unfortunately, in most studies, shellfish allergy prevalence was assessed using self-reported questionnaire-based methods, and there are limited number of studies with an assessment using sensitization (SPT and sIgE), and very few studies have established the prevalence of shellfish allergy using the gold-standard challenge criteria. Moreover, studies for isolated molluscan allergy prevalence are scarce, and, except a few, in all of them, self-reported questionnaire-based methods are used. The reason for this is the huge diagnostic gap for molluscan allergy, and this gap may result in an underestimation of the prevalence of an isolated molluscan allergy. In addition, an existing gap is also in the more comprehensive epidemiology of the severity of manifestation of molluscan-induced allergies, particularly life-threatening anaphylaxis, which is necessary to alarm policy makers on the risk level of molluscan allergen ingestion. The FAO and WHO [83] rated crustacean shellfish with a higher proportion of anaphylaxis 3+ regions and Mollusca with a higher proportion of anaphylaxis 1+ regions. In addition, there is also a gap in the level of potency of shellfish allergens. For Crustacea, there are limited data only for shrimps, which are rated by the FAO and WHO with low potency (ED10 mg range > 100 mg protein, and ED50 mg range > 1000 mg protein), and there is no data for other crustaceans. There is no data for any molluscan shellfish.

Therefore, to obtain reliable prevalence, severity, and potency data, particularly for molluscan allergy, further studies incorporating standardized confirmatory allergy testing are urgently needed. For this, standardized diagnostic tools and a wide repertoire of shellfish allergens (extracts and allergen components), particularly for molluscan species, is a prerequisite.

**Diagnostic gaps**: Table 1 and Table 2 show that for the top 5 economically most relevant species/genera of each group, only for a few of them, extracts/and or allergen components for allergen diagnostics are commercially available. This is particularly pronounced for species/genera for which most production is in Asia, especially China. This clearly demonstrates that the main global manufacturers of allergy diagnostics tools are mainly focused on patients from Europe and North America. Moreover, even for many species for which Europe and North America are the main producers/consumers, allergen extract/components are commercially unavailable. The main consequence of these gaps is that diagnostic tools for shellfish allergies are mainly based on shellfish extracts or even mixtures of shellfish extracts, instead of allergen components. As these extracts are not standardized and may lack a sufficient amount and diversity of important shellfish allergens, shellfish allergy diagnostics are not quite reliable. Furthermore, false negative testing results can put patient’s lives at risk. For the top economically relevant molluscan shellfish, there are only several extracts available for SPT or within in vitro diagnostics tools. The most critically missing components of the diagnostic repertoire are extracts from the group of clams, cockles, and arkshells, with *Ruditapes philipinarum* as the most striking example. Furthermore, the low sequence similarity between TPMs of flat oyster (*Ostrea edulis*) and cupped oyster (*Crasosstrea gigas*) should be taken into account and further examined particularly as *Ostrea edulis* is a component of in vitro diagnostic tools, while *Crasosstrea gigas* is a component of in vivo diagnostic tools (Table 2).

This is of particular importance for patients monosensitized to molluscan shellfish or to specific molluscan species, where diagnosis using current tools could be false negative, putting these patients at risk. This is also important for patients monosensitized to crustacean shellfish, with the aim of reducing the unnecessary avoidance of mollusks. Therefore, for the precise and unambiguous diagnostics of shellfish allergy and personalized recommendations, expanding the repertoire of allergen components from one species, as well as expanding the repertoire of different species with their allergen components, particularly the most relevant species, is urgently needed. For this identification of new candidate allergens, confirmation of their IgE reactivity and their approval by the WHO/IUIS is a prerequisite. In addition, standardized immunotherapy preparations using recombinant allergen components are essential for developing consistent immunotherapy protocols.

**Allergenicity gaps**: For the top economically relevant species, there are several gaps. Firstly, for most species or groups, there are **no allergens** approved by the WHO/IUIS, or there is only **one allergen**. Secondly, for many species, even nonregistered IgE-reactive allergens were not described yet. This gap is again particularly great for molluscan shellfish. Table 2 shows that allergens are registered for only five economically relevant Mollusca (mostly TPMs). Although for several species, nonregistered TPMs and other allergen IgE reactivity were documented, and for the top 5 economically relevant molluscan shellfish species, no allergens have been described yet. Within Mollusca shellfish, the most economically relevant species of oysters (*C. gigas*) are well characterized at the molecular level. All top 5 freshwater mollusk species do not have a single allergen described, nor available sequences for the top 3 species. For mussels, scallops, and clams, there are only several non-registered allergens with IgE reactivity and no registered allergens. Squids have only one registered and several non-registered allergens with IgE reactivity described. In contrast to Crustacea shellfish (Table 1), there are only several recombinant allergens currently commercially available, and none of them (except non-registered allergens Ven ga 1 and Uro du 1) are present within diagnostic tools (Table 2). Moreover, although many recombinant allergens currently are commercially available, they are not present within diagnostics tools, particularly allergens other than TPM. Other than TPM, only the top shrimp and prawn (white leg shrimp and giant tiger prawn) species, crab (green mud crab), and freshwater crustacean (red swamp crayfish) allergens are well described at the molecular level (Table 1). However, for a few species (such as queen crab and kuruma shrimp/*Marsupenaeus japonicus*), several allergens other than TPM have demonstrated IgE reactivity. The data presented in Table 1 clearly demonstrate that for the most economically relevant crustacean shellfish, not only that allergens approved by the WHO/IUIS are still missing, but for many of them, allergenicity was not sufficiently investigated. Therefore, for the development of reliable diagnostic tools, the faster and more comprehensive identification of new candidate allergens, particularly from the most relevant species and for molluscan species, and their testing and subsequent approval by the WHO/IUIS are needed. For the identification of new potential allergens and their testing for IgE reactivity eligible by the WHO/IUIS, recombinant allergens are necessary, taking into account difficulties related to the purification and standardization of natural allergens. Consequently, obtaining recombinant allergens requires available gene or protein sequences.

**Genomic/proteomic gaps**: For many species, there is a limited number of available gene or protein sequences of potential allergens, not only for TPM but also sequences of other potential allergens, such as AK, SCBP, etc. For molluscan species, sequences of potential allergens are very scarce, particularly for freshwater Mollusca, marine Gastropoda, and marine Cephalopoda shellfish (Table 2). For example, there are available protein sequences of TPM for only about 10 marine Gastropoda, or Cephalopoda, shellfish species, including unreviewed sequences. Similarly, for paramyosin, as the second relevant allergen in Mollusca, there are protein sequences from only several Gastropoda and Cephalopoda shellfish species. For flat oyster, *Ostrea edulis* (a frequent component of in vitro diagnostic tools), only a partial, unreviewed, sequence of TPM exists.

For economically highly relevant species of Chilean mussels (0.5 billion tones produced), jumbo flying squids (1 billion tones produced annually), Akiami paste shrimp *Acetes japonicus* (0.3 million tons produced annually*),* Blood cockle *Tegillarca granosa* (0.4 billion tones produced annually), Peruvian calico scallop, *Argopecten purpuratus,* Argentine shortfin squid*,* and *Illex argentinus*, there are no in vitro diagnostic tools available, registered allergens, or sequence information on the most common allergen of shellfish, tropomyosin (Table 1 and Table 2).

For research on gene or protein sequences of potential shellfish allergens, the list of the most relevant shellfish species is a prerequisite to direct this research. Thus, part of this review is focused on the global production, supply, and consumption of shellfish, providing an overview of the relevant shellfish species.

A very recent publication is a nice example of significant progress in the allergen profile of relevant shellfish species, which is crucial for developing CRD-based tests and preventive immunotherapy based on molecular allergens [84]. Based on the assembled P. clarkii (crayfish) genome using both long-read and short-read sequencing data, this study identified a total of 11 putative allergen groups, including all isoforms or homologs for each allergen group based on the genome, identified 2 previously unknown allergens (pPro c 3.0301 and pPro c 6.0201) with IgE reactivity of E reactivity of 33.3% and 20%, respectively, and additionally identified 3 putative allergens through 2D mass spectrometry. This comprehensive approach could be applied to other relevant shellfish species and may be particularly useful for relevant molluscan species.

In summary, the most urgent research on genomic, proteomic, allergenicity, and improvements in diagnostic and immunotherapy tools is necessary for the identified species. Progress in these aspects would allow more comprehensive and more reliable prevalence, severity, and potency data to be obtained, which in turn would be convincing for policy makers to introduce regulations on shellfish allergen labeling worldwide. Finally, reliable unequivocal diagnostics of shellfish allergy and shellfish allergen labelling together would reduce the risk of shellfish-induced allergic reactions and anaphylaxis. This is of particular importance for still undefined molluscan allergy resulting in allergic risks associated with the consumption of molluscan shellfish. This review, providing lists of the most economically relevant shellfish species, may serve as a guide not only for directions of research needed, but also for clinicians, diagnostic and immunotherapy tool manufacturers, and for policy makers.

## 8. Conclusions

Despite the growing popularity of shellfish, improvements in diagnostics and the better standardization of shellfish allergenic extracts is a very challenging task. The great diversity of shellfish species consumed is an obvious obstacle. Furthermore, the contents, concentrations, and ratios of the individual allergens vary greatly depending on a large variety of factors. Allergens frequently occur as different isoforms in varying quantities, and the presence of proteases in allergen extracts results in allergen degradation, contributing to heterogeneity in allergenic extracts [85]. Therefore, for more reliable shellfish allergy diagnosis, tools should be based on the implementation of recombinant allergen molecules and their mixtures that are defined regarding purity and biological activity. The prerequisite for the wide implementation of recombinant allergen molecules of shellfish is an investigation of allergen molecules in, at least the most frequently and/or widely consumed species, as well as the availability of their protein sequences.

Shellfish crustacean species are taxonomically highly related, belonging to the same order, Decapods, resulting in high sequence homology (>90%) of pan-allergen TPM. In contrast, molluscan shellfish belong to different classes (Bivalvia, Cephalopoda, and Gastropoda) resulting in much lower sequence homology (>70%) of TPM. Moreover, even mollusks belonging to the same order have TPM sequence homology <90%, e.g., only mollusks belonging to the same genus have sequence TPM homology >90%. The most striking example are oysters, cupped oysters, and flat oysters’ TPM, which share only about 55% homology. This implies that by using a recombinant TPM allergen from one Crustacea species, allergy to other crustacean shellfish can be more reliably diagnosed than by using recombinant TPM from one Mollusca species to diagnose allergy to other molluscan shellfish. Furthermore, regarding the sensitization rates to TPM, studies do not show that 100% of patients react to it, meaning that additional allergens are necessary for a precise diagnosis. The identifications of TPM, AK, and SCBP in several shellfish species as allergens are all important contributions, but additional allergens are required for the development of a complete set of reagents for component-resolved diagnosis and the exploration of novel vaccination strategies, particularly in Mollusca. Based on the WHO/IUIS allergen registry, the most comprehensive allergen profile exists from black tiger shrimp, *P. monodon*, comprising nine registered allergens, including TPM, AK, MLC2, SCP, Tn, Hc, TIM, FABP, and GP. The number of bivalve species known to be exploited by humans for food is about 800 [44], while only 5 Bivalvia allergens are registered by WHO/IUIS from only 3 species. Moreover, in the UniProt database, there are TPM sequences from about only 30 Bivalvia species.

Our review provided a comprehensive analysis of shellfish allergens in relation to the abundance of the food source on the global market. We have shown that the production of shellfish, such as Mollusca, is the overweight production of Crustacea globally, while lagging in view of not only the molecular characterization of allergens, but also the availability of in vivo and in vitro diagnostic tools based and molecular information on potential allergens. There are economically highly relevant species, present on the market, that are not presented in diagnostic tools. Therefore, the sensitization rate to those species cannot be known. as also other factors such as food processing methods, raw consumption, per capita intake, cultural dietary habits, and occupational exposure can also influence the allergy risk. Furthermore, the regulation of food allergen labeling in a wide range of countries affected by shellfish/molluscan allergies is inconsistent with the supply/capita of the shellfish.

Within the group of Mollusca, particular attention should be given to Cephalopoda, because of their dominance of the global trade market and rather minimal knowledge of causative allergens. Cephalopodes trades (squids being on top) represent 60% of the global production of shellfish, while only 1 allergen of Cephalopodes (Tod p 1.0101 from Japanese flying squid) out of 50 allergenic shellfish allergens is included so far in the WHO/IUIS. Of 52 non-registered proteins with IgE reactivity in Mollusca, only 9 are described in Cephalopoda (8 TPMs and 1 paramyosine), being candidates for future approval by the WHO/IUIS. Moreover, there are only five available protein sequences of TPM for Cephalopoda shellfish species, including unreviewed sequences. In diagnostic tools, Cephalopoda are represented only with recombinant Tod p 1 and few extracts (*Loligo* spp., Octopus vulgaris, Todarodes pacificus extract, Sepia officinalis).

The scarcity of molecular studies on Mollusca and Cephalopodes allergens leads to the absence of a reference for precise allergy diagnostics and accurate allergen immunodetection. In view of the widespread high consumption and allergy prevalence of Mollusca shellfish, this would be a hazard to the health of large populations. Thus, more systematic research studies of molluscan allergens are essential for the future development of diagnostic tools and improvement of food legislation and safety. The knowledge gaps identified through our study may represent a useful guide for further research and improvements of diagnostic tools and the better management of shellfish allergies.

## Figures and Tables

**Table 1:** Alignment of major economically relevant species/genera/families/orders of crustacean shellfish with the information on existing shellfish allergens (WHO/IUIS and non WHO/IUIS), cross-reactivity of TPM (if PubMed information was available) with registered TPM, and availability of any diagnostic tools (in vivo*,* in vitro) or pure component (allergen) for *in-house* allergenicity testing. For entries such as the group of species (genus, family, etc.), % homology is presented for all species of a specified group with the available complete sequence in UniProt. Details are shown in data available at https://hdl.handle.net/21.15107/rcub_cherry_7008, date accessioned 10 January 2025).

Common Name/Latin Name	Producers	Production [LWT]	WHO/IUIS Allergens	Non-WHO/IUIS IgE Reactive Allergens	TPM as Allergen or Putative Allergen (% Homology with Shellfish TPM Allergens Approved by WHO/IUIS)	Commercially Available Extract or Allergen Component for In Vivo or In Vitro Diagnosis
**Freshwater crustaceans**						
Red swamp crayfish/*Procambarus clarkii*	China (96.92%)	**2,717,183.83**	Pro c 1 (TPM), Pro c 2 (AK), Pro c 5 (MLC), and Pro c 8 (TIM)	Pro c 210 kDa (Ferritin), Pro c 4 (sarcoplasmic calcium-binding protein, SCBP), pPro c 3.0301 (MLC2), pPro c 6.0201(troponin C)	**>**91.9% with all crustacean (with **Hom a 1 98.24%**) and >60.56% with all molluscan (with Hal l 1 1 63.38%)	f178 *Procambarus clarkii* extract; rProc 1, rProc 2, rProc 5, rProc 8
Chinese mitten crab *Eriocheir sinensis*	China (99.99%)	**833,313.75**	Eri s 2 (ovary development related protein)	Eri s 1 (TPM), Eri s 4 (SCBP)	>91.55% with all crustacean (with **Scy p1 99.65%**) and >60.56% with all molluscan (with Tod p 1, Cra g 1 and Hal l 1 63.0.3%)	*Eriocheir sinensis* extract**; rEri s 2**
Giant river prawn *Macrobrachium rosenbergii*	China (52.24%), Bangladesh (15.48%), Thailand (13.25%)	**327,827.88**	Mac r 1 (TPM), Mac r 2 (AK)	Mac r HC (hemocyanin)	>92.25% with all crustacean (with **Cra c 1 99.65%**) and >61.27% with all molluscan (with Tod p 1, Cra g 1 and Hal l 1 63.38%)	**rMar c 1**
Oriental river prawn *Macrobrachium nipponense*	China (100%)	**273,493.50**		TPM, MLC	>91.55% with all crustacean (with **Hom a 1 98.24%**) and >60.92% with all molluscan (with Hal l 1 64.08%)	**rTPM**, **rMLC**
Freshwater prawns, shrimps nei *Palaemonidae*	China (63.60%)	**67,297.23**	Exo m 1 (TPM) from *Exopalaemon modestus*; Mac r 1 (TPM), and Mac r 2 (AK) from *Macrobrachium rosenbergii*	Mac r HC (hemocyanin) from *Macrobrachium rosenbergii*	All homologies listed for Macrobrachium rosenbergii and *Macrobrachium nipponense* TPM *Macrobrachium lanchesteri*: >92.25% with all crustacean (with Mac r 1 100%) and >61.27 with all molluscan (with Cra g 1, Hal l 1 and Tod p 1 with 63.38)	**rExo m1, rMac r 1**
**Crabs, sea-spiders**						
Gazami crab *Portunus trituberculatus*	China (95.48%)	**476,026.45**		Por tr 1 (TPM)	>91.2% with all crustacean (with **Scy p 1 99.65%**) and >60.56% with all molluscan (with Tod p 1, Cra g 1 and Hal l 1 63.03%)	*Portunus trituberculatus* extract
Blue swimming crab *Portunus pelagicus*	Indonesia (39.58%), China (27.26%), Philippines (12.81%)	**251,440.12**	Por p 1 (TPM)	Por p 2 (AK)	>91.9% with all crustacean (with **Pan s 1 98.54%**), >60.21% with all molluscan (with Tod p 1 and Hal l 1 63.03%)	**rPor p 1**
Green mud crab *Scylla paramamosain*	China (100%)	**152,065.00**	Scy p 1 (TPM), Scy p 2 (AK), Scy p 3 (MLC), Scy p 4 (SCBP), Scy p 8 (TPI), Scy p 9 (Filamin C)		>91.55% with all crustacean (with **Hom a 1 97.18%**) and >60.56% with all molluscan (with Tod p 1, Cra g 1 and Hal l 1 63.03%)	rScy p 1 rScy p 2, rScy p 3, rSci p 4, rScy p 8, rScy p 9
Indo-Pacific swamp crab *Scylla serrata*	Viet Nam (62.29%), Philippines (20.34%), Indonesia (14.00%)	**130,276.78**		Scy s 1 (TPM), Scy s 2 (AK)	>91.55% with all crustacean (with **Scy p 1 100%**) and >60.56% with all molluscan (with Tod p 1, Cra g 1 and Hal l 1 63.03%)	
Queen crab *Chionoecetes opilio*	Canada (63.16%)	**121,643.2**		Chi o 1 (TPM), Chi o 2 (AK), Chi o 4 (SCBP), Chi o 6 (Troponin C), Chi o alpha_Actin (alpha actin), Chi o SERCA (SERCA)	>87.96% with all crustacean TPM allergens (with **Scy p 1 94.72%**) and >59.15% with all molluscan (with Tod p 1, Cra g 1 and Hal l 1 61.27%)	f23 *Chionoecetes spp extract*; **rChi o 1, rChi o 6**
**King crabs, squat-lobsters**						
Red king crab *Paralithodes camtschatica*	Russian Federation (92.66%)	**28,911.00**	Para c 11 (mitochondrial malate dehydrogenase)	Para c 1 (TPM)	>90.14% with all crustacean (with **Hom a 1 98.24%**) and >59.51% homology with all molluscan TPM allergens (with Tod p 1 63.73%)	*Paralithodes camtschatica* extract
Blue king crab *Paralithodes platypus*	Russian Federation (100%)	**8365.00**				
Southern king crab *Lithodes santolla*	Chile (67.76%), Argentina (32.22%)	**6630.73**				
Carrot squat lobster *Pleuroncodes monodon*	Chile (100%)	**6176.00**				
Blue squat lobster *Cervimunida johni*	Chile (100%)	**3114.00**				
**Lobsters**						
St.Paul rock lobster *Jasus paulensis*	French Southern Terr (100%)	**219,709.00**				
American lobster *Homarus americanus*	Canada (63.37%), USA (36.62%)	**166,815.04**	Hom a 1 (TPM), Hom a 3 (MLC), Hom a 6 (troponin C)	Hom a 4 (SCBP)	>92.96% with all crustacean (with **Pan s 1 98.91%**) and >60.92% with all molluscan (with Hal l 1 63.73%)	F20 *Homarus americanus* extract, rHom a 1, rHom a 3, rHom a 6
Norway lobster *Nephrops norvegicus*	United Kingdom (59.21), Ireland (11.80%), Denmark (9.99%)	**54,228.33**		Nep n DF9 (unknown)		
Caribbean spiny lobster *Panulirus argus*	Brazil (24.90%), Bahamas (22.31%), Cuba (12.24%)	**29,316.95**				
Tropical spiny lobsters nei *Panulirus* spp.	Indonesia (25.56%), Nigeria (18.45%), Mexico (12.70%)	**27,131.16**	Pan s 1 (TPM) from *Panulirus stimpsoni*	Pan ho 1 (TPM) from *Panulirus homarus*; Pan j 1 (TPM) from *Panulirus japonicus*	TPM *Panulirus stimpsoni*: >92.34 with all crustacean (with Hom a 1 98.91) and >60.58 with all molluscan (with Cra g 1 and Hal l 1 62.77)	f71 *Palinurus elephas extract*; f 304 *Palinurus* spp. extract; F71 *Palinurus vulgaris* extract; **rPan s 1**
**Shrimps, prawns**						
Whiteleg shrimp *Penaeus vannamei/Litopenaeus vannamei*	China (31.15%), India (15.71%), Ecuador (14.06%)	**6,348,621.71**	Lit v 1 (TPM), Lit v 2 (AK), Lit v 3 (MLC), Lit v 4 (SCBP), Lit v 13 (fatty acid-binding protein)	Lit v HC (Hemocyanin)	>92.61% with all crustacean (with **Pen m 1 and Pen a 1 100%**) and >60.92% with all molluscan (with Hal l 1 63.38%)	*Litopenaeus vannamei* extract; r**Lit v 1, rLit v 2, rLit v 3, rLit v 4**
Giant tiger prawn *Penaeus monodon*	Viet Nam (30.14%), Indonesia (16.84%), China (11.84%)	**883,686.29**	Pen m 1 (TPM), Pen m 2 (AK), Pen m 3 (MLC), Pen m 4 (SCBP), Pen m 6 (troponin C), Pen m 7 (hemocyanin), Pen m 8 (TPI), Pen m 13 (cytoplasmic fatty acid-binding protein), Pen m 14 (glycogen phosphorylase-like protein)		>92.61% with all crustacean (with **Lit v 1 and Pen a 1 100%**) and >60.92% with all molluscan (with Hal l 1 63.38%)	f24 *Penaeus monodon* extract; r**Pen m 1, rPen m 2, rPen m 3, rPen m 4, rPen m 6, rPen m 7, rPen m 8, rPen m 14**
Natantian decapods nei *Natantia*	China (25.23%), Viet Nam (20.47%), India (25.20%)	**758,903.87**	All allergens listed for *Penaeus* spp., Met e 1 (TPM) from *Metapenaeus ensis*; Cra c 1 (TPM), Cra c 2 (AK), Cra c 4 (SCBP), Cra c 5 (MLC), Cra c 6 (troponin C), and Cra c 8 (TIM) from *Crangon crangon*; Pan b 1 (TPM) from *Pandalus borealis*	All allergens listed for *Penaeus* spp.; Pan e 1 (TPM) from *Pandalus eous*; Sol me 1 (TPM) from *Solenocera melantho*;	All listed homologies of *Penaeus* spp. TPM *Pandalus borealis*: >92.25% with all crustacean (with **Mac r 1 98.94%**) and >61.62% with all molluscan (with Hal l and Tod p 1 63.73%) TPM *Pandalus eous*: >92.25% with all crustacean (with **Pan b 1** 100%) and >61.62% with all molluscan (with Hal l 1 and Tod p 1 63.73%), TPM *Crangon crangon*: >92.25% with all crustacean (with **Mac r 1** 99.65%) and >61.62% with all molluscan (with Cra g 1, Hal l 1 and Tod p 1 63.38%)	All recombinant allergen extracts and components listed for *Penaeus* spp.; F241 *Pandalus borealis* extract, F23 *Crangon crangon* extract; *Parapenaeus longirostris* extract; **rCra c 1, rCra c 2, rCra c 4, rCra c 5, r Cra c 6, rCra c 8, natural shrimp TPM**
Akiami paste shrimp *Acetes japonicus*	China (94.75%)	**379,695.95**		TPM	Non-available TPM sequence	
Penaeus shrimps nei *Penaeus* spp.	China (39.62%), Peru (11.52%), Cameroon (10.76%)	**295,115.19**	Lit v 1 (TPM), Lit v 2 (AK), Lit v 3 (MLC), Lit v 4 (SCBP), and Lit v 13 (fatty acid-binding protein) from *Litopenaeus vannamei* (syn. *Penaeus vannamei*); Mel l 1 (TPM) from *Melicertus latisulcatus* (syn. *Penaeus latisulcatus*); Pen a 1 (TPM) from *Farfantepenaeus aztecus* (syn. *Penaeus aztecus*); Pen i 1 (TPM) from *Farfantepenaeus indicus* (syn. *Penaeus indicus*); Pen m 1 (TPM), Pen m 2 (AK), Pen m 3 (MLC), Pen m 4 (SCBP), Pen m 6 (troponin C), Pen m 7 (hemocyanin), Pen m 8 (TPI), Pen m 13 (Cytoplasmic fatty acid-binding protein) and Pen m 14 (glycogen phosphorylase-like protein) from *Penaeus monodon*;	Fen me 2 (AK) and Fen me 4 (SCBP) from *Fenneropenaeus merguiensis* (syn. P*enaeus merguiensis*); Lit v HC (Hemocyanin) from *Litopenaeus vannamei* (syn. Penaeus vannamei); Mar j 1 (TPM), Mar j 4 (SCBP), Mar j Enolase (enolase) and Mar j PK (pyruvate kinase) from *Marsupenaeus japonicas* (syn. *Penaeus japonicas*); Met j 2 (AK) from *Metapenaeus joyneri*,(syn. *Penaeus joyneri*); Pena o 1 (TPM) from *Penaeus orientalis*;	All homologies listed for *Penaeus monodon*, *Penaeus vannamei* TPM *Penaeus chinensis*: >92.61% with all crustacean (with P**en m 1**, **Pen a 1** and **Lit v 1** 100%) and >60.92% with all molluscan (with Hal l 1 63.38%), *Penaeus aztecus* TPM: >92.61% with all crustacean (with **Pen m 1** and **Lit v 1** 100%) and >60.92% with all molluscan (with Hal l 1 63.38%), *Metapenaeus ensis* TPM: >92.34% with all crustacean (with Pen m 1, Pen a 1 and Lit v 1 99.64%) and >60.22% with all molluscan (with Cra g 1 and Hal l 1 62.41%) *Penaeus japonicus* TPM: >92.61% with all crustacean (with Pen m 1, Pen a 1 and Lit v 1 100%) and >60.92% with all molluscan (with Hal l 1 63.38%) *Penaeus latisulcatus* TPM: >91.55% with all crustacean (with Pan b 1 95.42%) and >61.62% with all molluscan (with Cra g 1, Hal l 1 and Tod p 1 64.08%) *Penaeus merguiensis* TPM: >92.61% with all crustacean (with Pen m 1, Pen a 1 and Lit v 1 100%) and >60.92% with all molluscan (with Hal l 1 63.38%)	f24 *Penaeus monodon* extract; *Litopenaeus vannamei* extract; F34 *Farfantepenaeus aztecus*; rLit v 1, rLit v 2, rLit v 3, rLit v 4, rMel l 1, rPen a 1, rPen i 1, rPen m 1, rPen m 2, rPen m 3, rPen m 4, rPen m 6, rPen m 7, rPen m 8, rPen m 14, rMar j 2

**Table 2:** Alignment of major economically relevant species/genera/families/orders of Mollusca shellfish with the information on existing allergens (WHO/IUIS and non WHO/IUIS), cross-reactivity of TPM (if PubMed information was available) with registered TPM, and availability of any diagnostic tools (in vivo*,* in vitro) or pure component (allergen) for *in-house* allergenicity detection. For entries such as group of species (genus, family, etc.), % homology was presented for all species of a specified group with the available complete sequence in UniProt.

Common Name/Latin Name	The Main Producers (% of Total Production)	Production [LWT]	Allergens	Non-IUIS Allergens with IgE Reactivity	TPM as Allergen or Putative Allergen (% Homology with Shellfish TPM Allergens Approved by WHO/IUIS)	Commercially Available Extract or Allergen Component
**Freshwater mollusks**						
Freshwater mollusks nei Mollusca	China (76.32%), Philippines (14.61%), Korea, Republic of (3.81%)	**212,045.18**			Homology presented for *Corbicula japonica* *Dreissena polymorpha* TPM: >71.73% with all molluscan (with **Hal l 1** 75.97%) and >55.31% with all crustacean (with Pan b 1 and Hom a 1 57.24%) *Biomphalaria glabrata* TPM (Uniprot ID P42636): >76.76% with all molluscan (with **Hal l 1** 82.39%) and >60.22 with all crustacean (with Pan b 1 and Hom a 1 61.62%)	
Chinese mystery snail *Cipangopaludina chinensis*	China (100%)	**98,420.00**				
Chinese pond mussel *Sinanodonta woodiana*	China (100%)	**54,628.00**				
Asian clam *Corbicula fluminea*	China (84.31%), Taiwan Province of China (15.69%)	**26,385.74**				
Japanese corbicula *Corbicula japonica*	Japan (93.51%), Russian Federation (6.48%)	**9560.00**			>72.18% with all molluscan (with **Hal l 77.11%**) and >55.11% with all crustacean (with Hom a 1, Scy p 1 and Pan b 1 57.39%)	
**Abalones, winkles, conchs**						
Sea snails *Rapana* spp.	China (100%)	**299,620.00**	Rap v 2 (paramyosin) from *Rapana venosa*			**rRap v 2**
Abalones nei *Haliotis* spp.	China (88.71%), Republic of Korea (9.49%)	**245,567.51**	Hal l 1 (*Haliotis laevigata x Haliotis rubra*), TPM Hal m 1 (*Haliotis midae* (Perlemoen abalone)), TPM	Hal d 1 (TPM) from *Haliotis diversicolor*, Hal di 1 (TPM) from *Haliotis discus*, Hal di PM (paramyosin) from *Haliotis discus*, Hal r 1 (TPM) from *Haliotis laevigata x Haliotis rubra*, Hal r 49 kDa (unknown protein) from *Haliotis laevigata x Haliotis rubra*	*Haliotis discus discus* TPM: >78.17% with all molluscan (with **Hal l 1** 99.65%) and >62.41% with all crustacean (with Cra c 1, Pan b 1 and Mel l 1 63.73%) *Haliotis diversicolor* TPM: >77.46% with all molluscan (with **Hal l 1** 98.94%) and >61.68% with all crustacean (with Cra c 1 and Mel l 1 63.03%) *Haliotis rufescens* TPM: >75.7% with all molluscan (with **Hal l 1** 96.13%) and >60.22% with all crustacean (with Cra c 1 and Pan b 1 61.97%) *Haliotis asinine* TPM: >76.06% with all molluscan (with **Hal l 1** 94.37%) and >60.22% with all crustacean (with Cra c 1, Pan b 1 and Mel l 1 61.62%) *Haliotis laevigata x Haliotis rubra* TPM: >77.82% with all molluscan (with **Cra g 1** 82.39%) and >62.41% with all crustacean (with Mel l 1 64.08%)	f935 *Haliotis japonica* extract; f346 *Haliotis* spp. Extract; **rHal l 1, rHal m 1, rHal di 1**
Gastropods nei Gastropoda	Mexico (34.56%)	**40,514.86**	All allergens listed for *Rapana* spp. and *Haliotis* spp.	**All allergens listed for *Rapana* spp. and *Haliotis* spp.,** Tur c 1 (TPM), Tur c PM (paramyosin) from Horned turban (*Turbo cornutus*)	All homologies listed for *Rapana* spp. and *Haliotis* spp. *Lottia gigantean* TPM: >75.7% with all molluscan (with **Hal l 1** 85.21%) and >62.04% with all crustacean (with Mel l 1 63.73%) *Elysia crispata* TPM: >50.18% with all molluscan (with **Hal l 1** 54.8%) and >40.18% with all crustacean (with Met e 1 46.62%) *Elysia chlorotica* TPM: >72.18% with all molluscan (with **Hal l 1** 84.45%) and >63.03% with all crustacean (with Pan b 1 64.08%) *Neptunea polycostata* TPM: >73.24% with all molluscan (with **Hal l 1** 77.46%) and >59.15% with all crustacean (with Mel l 1 61.62%)	All recombinant allergen extracts and components listed for ***Rapana* spp. and *Haliotis* spp.**, **rTur c 1**
Whelk Buccinum undatum	United Kingdom (49.46%), France (31.29%), Ireland (15.16%)	**38,276.59**				
Stromboid conchs nei *Strombus* spp.	Nicaragua (50.36%), Belize (12.27%), Turks and Caicos Is. (6.47%)	**32,588.57**				
**Oysters**						
Cupped oysters nei *Crassostrea* spp.	China (99.33%)	**5,858,347.36**	Cra g 1.01 (TPM) Cra g 1.02 (TPM) *Crassostrea gigas* (Pacific cupped oyster); Cra a 1 (TPM) Cra a 2 (AK) Cra a 4 (SCP) Portyuguese *Crassostrea angulata* Sac g 1 (TPM) from *Saccostrea glomerata* (syn. *Crassostrea glomerata*)		All homologies for *Crassostrea gigas* and *Crassostrea virginica*	**rCra a 4, rCra g 1, rSac g 1** f23 *Crassostrea virginica* extract
Pacific cupped oyster *Crassostrea gigas*	Korea, Republic of (50.60%), Japan (24.29%), France (13.06%)	**652,011.94**	**Cra g 1.01 (TPM)** **Cra g 1.02 (AK)**		>71.83% with all molluscan (with **Cra a 1 94.74%**) and >60.95% with all crustacean (with Pan s 1, Hom a 1, and Mel l 1 66.23%)	**rCra g 1**
American cupped oyster *Crassostrea virginica*	USA (75.07%), Mexico (20.51%), Canada (4.42%)	**226,485.41**			>80.77% with all molluscan (with **Cra g 1 88.24%**) and >65% with all crustacean (with Pen m 1, Pen a 1, Lit v 1, Met e 1, Pan b 1, Mac r 1 Pan s 1, and Mel l 1 68.78%)	f23 *Crassostrea virginica* extract;
Slipper cupped oyster *Crassostrea Iredalei*	Philippines (100%)	**40,799.75**				
Flat and cupped oysters nei (flat—Ostrea) Ostreidae *Ostrea edulis*	Australia (100%)	**8051.32**	All allergens listed for *Crassostrea* spp.		All listed homologies of *Crassostrea virginica*, *Crassostrea gigas*, and *Crassostrea rhizophorae* *Saccostrea glomerata* TPM: >75% with all molluscan (with **Cra a 1** and **Cra g 1 96.48%**) and >60.21% with all crustacean (with Cra c 1, Pan b 1 and Mel l 1 61.62%)	F177 *Ostrea edulis* extract
**Mussels**						
Sea mussels nei Mytilidae	China (78.07%), Spain (19.13%)	**1,062,460.81**		Myt e 1 (TPM) from *Mytilus edulis*; Myt g PM (paramyosin) from *Mytilus galloprovincialis*	All listed homologies of *Mytilus edulis* and *Perna viridis* TPM *Mytilus galloprovincialis*: >70.77% with all molluscan (with **Sac g 1 80.99%**) and >56.2% with all crustacean (with Cra c 1 and Mel l 1 58.45%)	rMyt g 1, r Myt g PM, rMyt e 1 f 37 *Mytilus edulis* extract
Chilean mussel *Mytilus chilensis*	Chile (100%)	**425,833.00**				
Blue mussel *Mytilus edulis*	France (29.23%), Denmark (17.64%), Netherlands (Kingdom of the) (17.41%)	**188,669.96**		Myt e 1 (TPM) from *Mytilus edulis*	>70.42% with all molluscan (with **Sac g 1 80.63%**) and >55.84% with all crustacean (with Cra c 1 and Mel l 1 58.1%)	f 37 *Mytilus edulis* extract; **rMyt e 1**
Green mussel *Perna viridis*	Thailand (44.28%), Indonesia (21.18%), Philippines (20.18%)	**117,603.10**		Per v 1 (TPM)	>69.37% with all molluscan (with **Sac g 1 80.63%**) and >54.74% with all crustacean (with Mel l 1 57.39%)	**rPerv1**
New Zealand mussel *Perna canaliculus*	New Zealand (100%)	**98,151.73**				
**Scallops, pectens**						
Scallops nei Pectinidae	China (99.75%)	**1,834,531.33**		Pat y 1 (TPM) from Yesso scallop (*Patinopecten yessoensis*)	Homology presented for *Patinopecten yessoensis* *Chlamys nipponensis akazara* TPM: >69.72% homology with all molluscan (with **Cra g 1 73.94%**) and >55.84% with all crustacean (with Mel l 1 58.8%) *Mimachlamys nobilis* TPM: >70.07% homology with all molluscan (with **Cra g 1 73.94%**) and >56.2% with all crustacean (with Hom a 1 58.8%) *Argopecten irradians* TPM: >69.72% homology with all molluscan (with **Cra g 1 and Sac g 1 74.3%**) and >54.74% with all crustacean (with Mel l 1 57.75%)	f 338 *Pecten* spp. extract; f328 *Chlamys varia* extract; f32 *Placopecten magellanicus* extract; f338 *Pecten maximus* extract
Yesso scallop *Patinopecten yessoensis*	Japan (93.88%), Russian Federation (5.10%)	**554,409.08**		Pat y 1 (TPM)	>69.37% homology with all molluscan (with **Cra g 1 73.94%**) and >55.47% with all crustacean (with Mel l 1 and Scy p 1 58.1%)	
American sea scallop *Placopecten magellanicus*	USA (73.99%), Canada (26.00%)	**220,861.24**				f32 *Placopecten magellanicus* extract
Peruvian calico scallop *Argopecten purpuratus*	Peru (96.55%)	**112,904.15**				
Great Atlantic scallop *Pecten maximus*	France (54.20%), United Kingdom (38.62%), Ireland (3.79%)	**72,447.55**				f338 *Pecten maximus* extract
**Clams, cockles, arkshells**						
Japanese carpet shell *Ruditapes philippinarum*	China (98.15%)	**4,358,635.76**		Ven ph 1 (TPM) (syn. *Venerupis philippinarum*)	>73.14% with all molluscan (with **Hal l 1 77.03%**), >57.51% with all crustacean (with Mel l 1 59.86%)	
*Sinonovacula constricta*	China (100%)	**859,651.00**		Sin c 1 (TPM)	>68.9% with all molluscan (with **Hal l 1 73.5%**) and >55.68% with all crustacean (with Hom a 1 58.3%)	
Blood cockle *Tegillarca granosa*	China (68.61%), Indonesia (18.74%), Thailand (7.47%)	**496,118.64**			>72.18% with all molluscan (with **Hal l 1 and Sac g 1 80.28%**) and >58.76% with all crustacean (with Cra c 1 and Pan b 1 60.56%)	
Clams, etc. nei Bivalvia	Korea, Dem. People’s Rep (49.98%), Japan (18.87%), Korea, Republic of (7.69%)	**124,038.51**		Ana br 1 (TPM) from *Anadara broughtonii*; Lut p 1 (TPM) from *Lutraria philippinarum*; Pin a 1 (TPM) from *Pinna atropurpurea*; Sin c 1 (TPM) from *Sinonovacula constricta*; Sol st 1 (TPM) from *Solen strictus*; Spi sa 1 (TPM) from *Spisula sachalinensis*; Tre ke 1 (TPM) from *Tresus keenae*; Ven ph 1 (TPM) from *Venerupis philippinarum*;	All homologies listed for *Tegillarca granosa*, *Ruditapes philippinarum*, *Sinonovacula constricta* *Anadara broughtonii* TPM: >71.83% homology with all molluscan (with **Sac g 1 79.93%**) and >58.76% with all crustacean (with Pan b 1 60.21%)	F10 *Mercenaria mercenaria* extract; F328 *Chlamys varia* extract;; F176 *Venus gallina* extract
Ocean quahog *Arctica islandica*	USA (99.99%)	**85,521.00**				
**Squids, cuttlefishes, octopuses**						
Jumbo flying squid *Dosidicus gigas* (genus: Dosidicus)	Peru (51.55%), China (42.02%), Chile (5.33%)	**1,004,277.76**				
Various squids nei Loliginidae, Ommastrephidae	China (62.51%), India (13.93%), Morocco (5.40%)	**542,466.49**	Tod p 1 (TPM) from *Todarodes pacificus*	All allergens listed for *Loligo* spp.; Omm b 1 (TPM) from *Ommastrephes bartramii*;	All listed homologies for *Loligo* spp. (*Loligo bleekeri* and *Ommastrephes bartramii*) TPM *Todarodes pacificus*: >75% with all molluscan (**with Hal l 1 80.99%**) and >62.04% with all crustacean (with Mel l 1 1 64.08%)	rTod p 1
Argentine shortfin squid *Illex argentinus*	Taiwan Province of China (32.61%), China (31.25%), Argentina (29.57%)	**447,091.58**				
Cephalopods nei Cephalopoda	Viet Nam (84.87%), China (10.51%), Magadascar (2.48%)	**423,596.21**	Tod p 1 (TPM) from *Todarodes pacificus*	All allergens listed for *Loliginidae*, *Ommastrephidae*; Ent d 1 (TPM) from *Enteroctopus dofleini*; Oct f 1 (TPM) and Oct f 2 (AK) from *Octopus fangsiao*; Oct l 1 (TPM) from O*ctopus luteus*; Oct v 1 (TPM) from *Octopus vulgaris*; Sep e 1 (TPM) from *Sepia esculenta*; Sep m 1 (TPM) from *Sepia madokai*	All listed homologies for *Loliginidae* and *Ommastrephidae* *Octopus bimaculoides* TPM: >75.7% with all molluscan (**with Tod p 1 91.2%**) and >62.77% with all crustacean (with Pan b 1 64.79%) *Sepioteuthis lessoniana* TPM: >75% with all molluscan (**with Tod p 1 96.48%**) and >61.68% with all crustacean (with Mel l 1 63.73%) *Sepia esculenta* TPM: >75.35% with all molluscan (**with Tod p 1 97.54%**) and >62.41% with all crustacean (with Mel l 1 and Scy p 1 64.44%)	**rTod p 1; F176 *Loligo* spp. extract; F819 *Octopus vulgaris extract*;** F108 *Todarodes pacificus* extract; F120 *Sepia officinalis*
Common squids nei *Loligo* spp.	Indonesia (61.46%), Thailand (19.60%), Philippines (13.98%)	**332,201.68**		Lol b 1 (TPM) from *Loligo bleekeri*; Omm b 1 (TPM) from *Ommastrephes bartramii* (syn. *Lologo bartramii*); Uro ed 1 (TPM) from *Loligo edulis*	*Loligo bleekeri* TPM: >76.41% with all molluscan (**with Tod p 1 92.96%**) and >60.58% with all crustacean (with Cra c 1, Mac r 1, Scy p 1 and Hom a 1 61.97%) *Ommastrephes bartramii* TPM: >74.65% with all molluscan (**with Tod p 1 96.13%**) and >61.68% with all crustacean (with Mel l 1 and Scy p 1 63.73%)	F176 *Loligo* spp. extract;

## Data Availability

Data on the cross-reactivity of tropomyosins are available from the Cherry repository of the University of Belgrade-Faculty of Chemistry, from the link https://hdl.handle.net/21.15107/rcub_cherry_7008, date accessioned 10 January 2025). Data on the nonregistered allergens of shellfish are available from the Cherry repository of the University of Belgrade-Faculty of Chemistry, from the link https://cherry.chem.bg.ac.rs/handle/123456789/7052, date accessioned 7 April 2025.

## Supplementary Materials

The following supporting information can be downloaded at: https://www.mdpi.com/article/10.3390/foods15101720/s1, Figure S1: (A) Daily animal protein supply per capita in selected world regions in 2021; (B) animal blue food production data from 2021; Table S1: Global production of shellfish in 2021; Table S2: Top 20 countries with the highest Crustaceas production in 2021; Table S3: Top 20 countries with the highest Mollusca ex. Cephalopoda production in 2021; Table S4: Top 20 countries with the highest Cephalopoda production in 2021; Table S5: Total global production of the most important crustacean shellfish in 2021; Table S6: Total global production of the most important molluscan shellfish in 2021; Table S7: Top 20 countries with the highest Cephalopoda export in 2021; Table S8: Top 20 countries with the highest Crustaceas export in 2021; Table S9: Top 20 countries with the highest Mollusca ex. Cephalopoda export in 2021; Table S10: Top 20 countries with the highest Cephalopoda import in 2021; Table S11: Top 20 countries with the highest crustacean import in 2021; Table S12: Top 20 countries with the highest Mollusca ex. Cephalopoda import in 2021; Table S13: Top 20 countries with the highest Crustacea, Mollusca (excluded Cephalopoda), and Cephalopoda supply quantity per capita per year in 2021; Table S14: Crustacean food allergens from the database of the World Health Organization and International Union of Immunological Societies (WHO/IUIS); Table S15: Molluscan food allergens from the database of the World Health Organization and International Union of Immunological Societies (WHO/IUIS); Table S16: Commercially available shellfish allergen extracts for SPT and in vitro allergy diagnostics from several producers; Table S17: Commercially available extract and allergen components within several singleplex and multiplex systems.
