# Supplementary material for: Critical Knowledge Gaps for Shellfish Allergies: Insights from Global Market Presence and Trade of Shellfish
Source: Foods. 2026 May 13;15(10):1720. doi: 10.3390/foods15101720 (PMC13206035; doi:10.3390/foods15101720)
Supplement: Supplementary file 1 [file foods-15-01720-s001.zip › foods-4247487-supplementary.pdf]

Supplementary material

Critical Knowledge and Diagnostic Gaps for Shellfish Allergies: Insights from Global Market Presence and Trade of shellfish

Dragana Stanic-Vucinic[1], Mirjana Radomirovic[1], Xuli Wu[2], Marija Stojadinovic[1] and Tanja Cirkovic Velickovic[1,3#]

Center of Excellence for Molecular Food Sciences, University of Belgrade – Faculty of Chemistry, Studentski trg 16, 11 000 Belgrade

School of Public Health, Health Science Center, Shenzhen University, Shenzhen, Guangdong Province 518060, PR China

Serbian Academy of Sciences and Arts, Knez Mihajlova 35, Belgrade, Serbia

Additional data availability:

Data on the cross-reactivity of tropomyosins are available from the Cherry repository of the University of Belgrade-Faculty of Chemistry, from the link: https://hdl.handle.net/21.15107/rcub_cherry_7008.

Data on the nonregistered allergens of shellfish are available from the Cherry repository of the University of Belgrade-Faculty of Chemistry, from the link:

https://cherry.chem.bg.ac.rs/handle/123456789/7052

Section S1. Introduction

A)

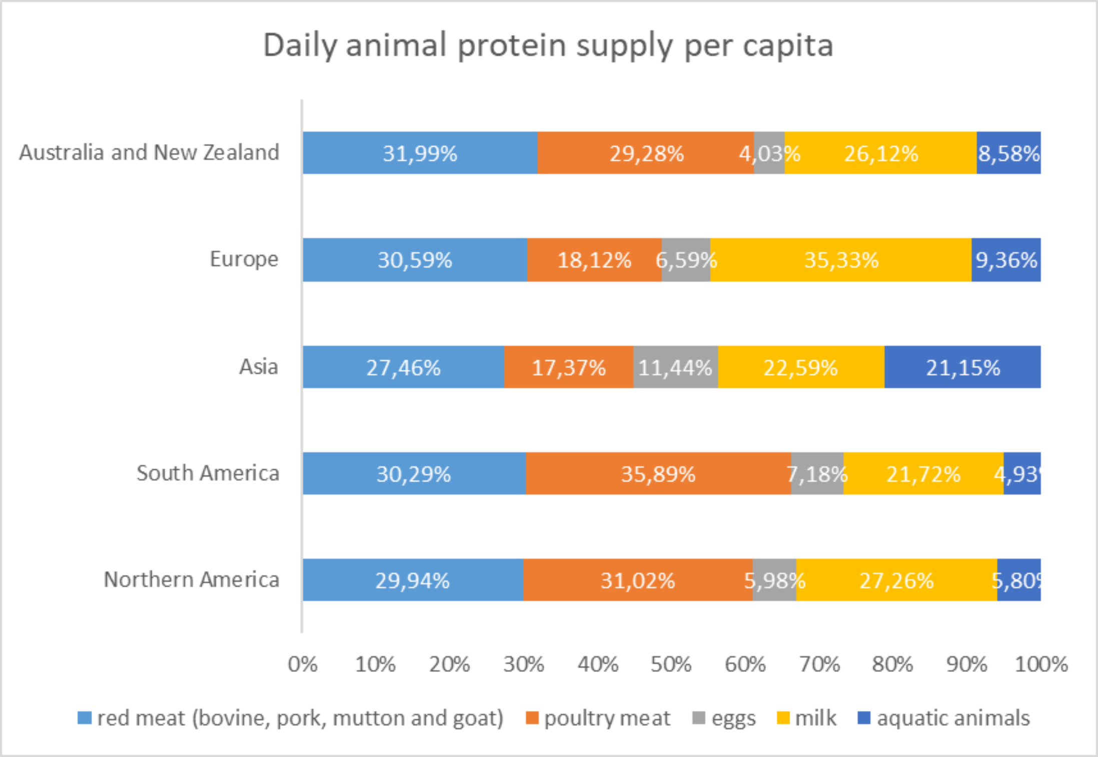

B)

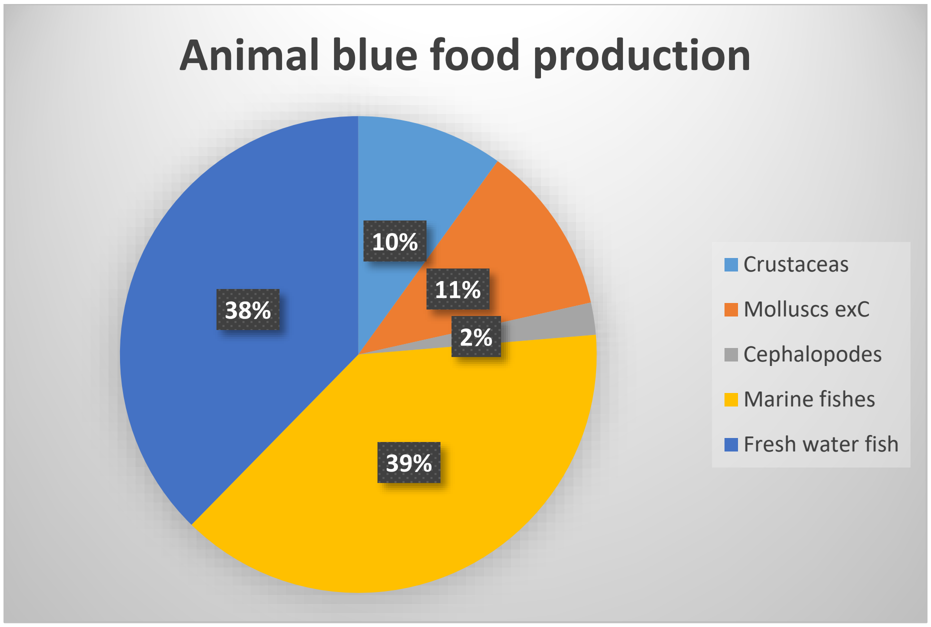

Figure S1. A) Daily animal protein supply per capita in selected world regions in 2021. Data source: Food and Agriculture Organization of the United Nations, FAOSTAT. Protein supply quantity (g/capita/day) for

the following sections was used in analysis: red meat (bovine meat, mutton and goat meat, pigmeat), poultry meat, eggs, milk (excluding butter), aquatic animals (freshwater fish, demersal fish, pelagic fish, marine fish other, crustaceans, cephalopods, mollusks and other); B) Animal blue food production data from 2021 mined from: https://www.fao.org/fishery/en/statistics/software/fishstatj

Section S2. Overview of global production and regulatory landscape

Section S2.1. Production of shellfish

Table S1. Global production of shellfish in 2021. The date are mined from FAO database (https://www.fao.org/fishery/en/statistics/software/fishstatj).

	FAO classes of shellfish	Tons of live weight	% of total Mollusca	% of total shellfish
Mollusca shellfish	Abalones, winkles, conchs	702,146.4	2.83	1.66
	Clams, cockles, arkshells	6,334,657.28	25.54	14.95
	Freshwater molluscs	402,202.02	1.62	0.95
	Miscellaneous marine molluscs	1,680,333.69	6.78	3.96
	Mussels	2,076,863.65	8.37	4.90
	Oysters	6,802,581.7	27.43	16.05
	Scallops, pectens	2,870,836.37	11.58	6.77
	Squids, cuttlefishes, octopuses	3,930,393.55	15.85	9.27
	Total	24,800,014.66	100	58.52
Crustacea shellfish	Shrimps, prawns	10,510,144.94	59.78	24.80
	Miscellaneous marine crustaceans	376,605.36	2.14	0.89
	Lobsters, spiny-rock lobsters	313,514.82	1.78	0.74
	King crabs, squat-lobsters	63,456.4	0.36	0.15
	Crabs, sea-spiders	1,949,065.38	11.09	4.60
	Freshwater crustaceans	4,368,580.54	24.85	10.31
	Total	17,581,367.44	100	41.48

Total shellfish (Tons of live weight) in 2021: 42,381,382.10

Table S2. Top 20 countries with the highest Crustaceas production in 2021. The data are mined from FAOSTAT database (https://www.fao.org/faostat/en/).

	Crustacea production	Kilotons	% of global production
1	**China, mainland**	7877.48	**47.61**
2	Indonesia	1502.73	9.08
3	India	1281.05	7.74
4	Viet Nam	1059.64	6.40
5	Ecuador	688.25	4.16
6	Thailand	533.15	3.22
7	Mexico	304.66	1.84
8	Norway	270.57	1.64
9	United States of America	256.93	1.55
10	Bangladesh	251.94	1.52
11	Canada	251.84	1.52
12	Argentina	218.1	1.32
13	Malaysia	176.75	1.07
14	Philippines	166.16	1.00
15	Myanmar	157.93	0.95
16	Russian Federation	141.28	0.85
17	Brazil	110.95	0.67
18	Peru	96.37	0.58
19	Republic of Korea	79.78	0.48
20	United Kingdom of Great Britain and Northern Ireland	74.46	0.45
	Global production	16547.35	
	Top 20 countries production (% of global production)		**93.67**

Table S3. Top 20 countries with the highest Mollusca ex. Cephalopoda production in 2021. The data are mined from FAOSTAT database (https://www.fao.org/faostat/en/).

	**Mollusca ex. Cephalopoda production**	**Kilotons**	**% of global production**
1	**China, mainland**	**15196.14**	**75.11**
2	Japan	700.74	3.46
3	United States of America	646.31	3.19
4	Republic of Korea	527.89	2.61
5	Chile	435.3	2.15
6	Viet Nam	345.98	1.71
7	Spain	239.03	1.18
8	France	205.68	1.02
9	Peru	155.99	0.77
10	Canada	155.68	0.77
11	Thailand	149.16	0.74
12	India	136.65	0.68
13	Indonesia	132.39	0.65
14	Italy	115.58	0.57
15	Philippines	107.79	0.53
16	New Zealand	104.34	0.52
17	Mexico	94.91	0.47
18	United Kingdom of Great Britain and Northern Ireland	79.02	0.39
19	China, Taiwan Province of	74.53	0.37
20	Democratic People's Republic of Korea	62.4	0.31
	Global production	20231.32	
	Top 20 contries prodution (% of global)		**97.20**

Table S4. Top 20 countries with the highest Cephalopoda production in 2021. The data are mined from FAOSTAT database (https://www.fao.org/faostat/en/).

	Cephalopoda production	Kilotons	% of global production
1	**China, mainland**	1002.02	**27.03**
2	Peru	539.18	14.55
3	Viet Nam	371.69	10.03
4	Indonesia	251.89	6.80
5	India	217.7	5.87
6	Japan	109.8	2.96
7	Republic of Korea	104.18	2.81
8	Thailand	98.37	2.65
9	Russian Federation	98.23	2.65
10	Argentina	96.3	2.60
11	Morocco	89.76	2.42
12	Malaysia	70.6	1.90
13	Chile	61.62	1.66
14	United States of America	53.94	1.46
15	Mexico	52.26	1.41
16	Philippines	52.18	1.41
17	Mauritania	49.23	1.33
18	China, Taiwan Province of	44.9	1.21
19	New Zealand	43.97	1.19
20	Myanmar	34.75	0.94
	Global production	3706.66	
	Top 20 contries prodution (% of global)		**92.88**

Table S5. Total global production of the most important Crustacean shellfish in 2021, including aquaculture and capture production. The species produced in > 1000 tones and their main producers are presented. The data are mined from FAO database (https://www.fao.org/fishery/en/statistics/software/fishstatj)

Common name	Latin name	Tons of live weight	The main producers (% of total production)
**Freshwater crustaceans**			
Red swamp crawfish	*Procambarus clarkii*	2,717,183.83	China (96.92 %)
Chinese mitten crab	*Eriocheir sinensis*	833,313.75	China (99.99 %)
Giant river prawn	*Macrobrachium rosenbergii*	327,827.88	China (52.24 %), Bangladesh (15.48 %), Thailand (13.25 %)
Oriental river prawn	*Macrobrachium nipponense*	273,493.50	China (100 %)
Freshwater crustaceans nei	Crustacea	80,988.72	Bangladesh (81.77 %), Russian Federation (6.19 %), Egypt (3.47 %)
Freshwater prawns, shrimps nei	Palaemonidae	67,297.23	China (63.60 %), Egypt (16.89 %), Bangladesh (11.78 %)
Siberian prawn	*Exopalaemon modestus*	49,080.50	China (100 %)
River prawns nei	Macrobrachium spp	9,202.20	Brazil (56.51 %), Mexico (38.84 %)
Euro-American crayfishes nei	Astacidae, Cambaridae	7,876.12	USA (62.63 %), Sweden (15.03 %), Spain (7.62 %)
**Crabs, sea-spiders**			
Gazami crab	*Portunus trituberculatus*	476,026.45	China (95.48 %)
Marine crabs nei	*Brachyura*	339,416.42	China (28.80 %), Viet nam (15.17 %), Mexico (11.30 %)
Blue swimming crab	*Portunus pelagicus*	251,440.12	Indonesia (39.58 %), China (27.26 %), Philippines (12.81 %)
Green mud crab	*Scylla paramamosain*	152,065.00	China (100 %)
Indo-Pacific swamp crab	*Scylla serrata*	130,276.78	Viet Nam (62.29 %), Philippines (20.34 %), Indonesia (14.00 %)
Queen crab	*Chionoecetes opilio*	121,643.20	Canada (63.16 %), USA (16.46 %), Russian Federation (11.93 %)
Portunus swimcrabs nei	Portunus spp	105,381.16	China (99.91 %)
Blue crab	*Callinectes sapidus*	74,978.71	USA (71.31 %), Mexico (19.83 %)
Tanner crabs nei	Chionoecetes spp	66,901.81	Russian Federation (64.11 %), Korea, Republic of (32.15 %)
Edible crab	*Cancer pagurus*	42,327.38	United Kingdom (57.97 %), Ireland (18.07 %), Norway (12.20 %)
Dungeness crab	*Cancer magister*	38,866.19	USA (75.01 %), Canada (24.99 %)
Clown crab	*Carpilius maculatus*	32,041.4.	Indonesia (100 %)
Orange mud crab	*Scylla olivacea*	31,465.71	Bangladesh (39.21 %), India (26.70 %), Indonesia (24.26 %)

Charybdis crabs nei	Charybdis spp	26,331.00	China (100 %)
Red snow crab	*Chionoecetes japonicus*	13,100.00	Japan (100 %)
Spinous spider crab	*Maja squinado*	9,638.29	France (83.19 %), Ireland (5.19 %), United Kingdom (4.96 %)
Jonah crab	*Cancer borealis*	5,020.00	USA (100 %)
West African geryon	*Chaceon maritae*	3,704.90	Namibia (80.97 %), Japan (14.59 %)
Mola rock crab	*Metacarcinus edwardsii*	3,645.00	Chile (100 %)
[Scylla spp]	Scylla spp	3,478.88	Thailamd (100 %)
Atlantic rock crab	*Cancer irroratus*	3,140.49	Canada (79.88 %), USA (20.12 %)
Velvet swimcrab	*Necora puber*	2,521.52	United Kingdom (78.19 %), Ireland (13.12 %)
Mediterranean shore crab	*Carcinus aestuarii*	2,487.92	Grece (85.97 %), Tunisia (12.26 %)
Periscope crab	*Podophthalmus vigil*	2,414.01	Indonesia (100 %)
Dana swimcrab	*Callinectes danae*	2,000.00	Brazil (100 %)
Green crab	*Carcinus maenas*	1,565.96	Portugal (29.18 %), Ireland (25.38 %), United Kingdom (31.46 %)
Black stone crab	*Menippe mercenaria*	1,059.51	USA (91.74 %)
Tanner crab	*Chionoecetes bairdi*	1,022.00	USA (100 %)
**King crabs, squat-lobsters**			
Red king crab	*Paralithodes camtschatica*	28,911.00	Russian Federation (92.66 %)
Blue king crab	*Paralithodes platypus*	8,365.00	Russian Federation (100 %)
Southern king crab	*Lithodes santolla*	6,630.73	Chile (67.76 %), Argentina (32.22 %)
Carrot squat lobster	*Pleuroncodes monodon*	6,176.00	Chile (100 %)
Blue squat lobster	*Cervimunida johni*	3,114.00	Chile (100 %)
Golden king crab	*Lithodes aequispina*	2,935.00	Russian Federation (100 %)
King crab	*Lithodes ferox*	2,743.00	USA (100 %)
Softshell red crab	*Paralomis granulosa*	2,621.00	Chile (100 %)
Right-handed hermit crabs nei	Paguridae	1,018.56.00	USA (99.75 %)
**Lobsters**			
St.Paul rock lobster	*Jasus paulensis*	219,709.00	French Southern Terr (100 %)
American lobster	*Homarus americanus*	166,815.04	Canada (63.37 %), USA (36.62 %)
Norway lobster	*Nephrops norvegicus*	54,228.33	United Kingdom (59.21), Ireland (11.80 %), Denmark (9.99 %)
Caribbean spiny lobster	*Panulirus argus*	29,316.95	Brazil (24.90 %), Bahamas (22.31 %), Cuba (12.24 %)
Tropical spiny lobsters nei	Panulirus spp	27,131.16	Indonesia (25.56 %), Nigeria (18.45 %), Mexico (12.70 %)

Australian spiny lobster	*Panulirus cygnus*	7,795.19	Austarlia (100 %)
Blunt slipper lobster	*Scyllarides squammosus*	5,170.42	Indonesia (100 %)
European lobster	*Homarus gammarus*	4,878.42	United Kingdom (65.03 %), France (12.65 %), Ireland (11.77 %)
Ornate spiny lobster	*Panulirus ornatus*	2,708.00	Tanzania (100 %)
Red rock lobster	*Jasus edwardsii*	2,552.74	New Zealand (100 %)
Mozambique lobster	*Metanephrops mozambicus*	2,085.50	South Africa (61.64 %), Mozambique (38.36%)
Southern rock lobster	*Jasus novaehollandiae*	1,818.98	Australia (100 %)
Lobsters nei	*Reptantia*	1,612.12	India (75.37 %), Viet Nam (20.78 %)
Longlegged spiny lobster	*Panulirus longipes*	1,128.58	Japan (97.47 %)
Cape rock lobster	*Jasus lalandii*	1,072.10	South Africa (90.67 %)
New Zealand lobster	*Metanephrops challengeri*	1,053.47	New Zealand (100 %)
**Shrimps, prawns**			
Whiteleg shrimp	*Penaeus vannamei/Litopenaeus vannamei*	6,348,621.71	China (31.15 %), India (15.71 %), Ecuador (14.06 %)
Giant tiger prawn	*Penaeus monodon*	883,686.29	Viet Nam (30.14 %), Indonesia (16.84 %), China (11.84 %)
Natantian decapods nei	Natantia	758,903.87	China (25.23 %), Viet Nam (20.47 %), India (25.20 %)
Akiami paste shrimp	*Acetes japonicus*	37,9695.95	China (94.75 %)
Penaeus shrimps nei	Penaeus spp	295,115.19	China (39.62 %), Peru (11.52 %), Cameroon (10.76 %)
Northern prawn / Northern shrimp	*Pandalus borealis*	248,817.54	Greenland (42.89 %), Canada (22.92), Norway (13.83 %)
Southern rough shrimp	*Trachysalambria curvirostris*	242,450.40	China (98.68 %)
Fleshy prawn/Chinese white shrimp	*Penaeus chinensis*	240,710.56	China (98.31 %)
Metapenaeus shrimps nei	Metapenaeus spp	223,814.45	Indonesia (68.53 %), Viet Nam (23.53 %)
Sergestid shrimps nei	Sergestidae	76,468.00	Malaysia (54.88 %), Thailand (33.69 %), Philippines (10.95 %)
Northern white shrimp	*Penaeus setiferus*	54,132.12	USA (98.28 %)
Kuruma prawn	*Penaeus japonicus*	47,686.87	China (93.42 %),
Banana prawn	*Penaeus merguiensis*	44,556.00	Indonesia (67.24 %), Thailand (21.72 %), Australia (8.05 %)
Northern brown shrimp	*Penaeus aztecus / Farfantepenaeus aztecus*	43,734.87	USA (73.32 %), Mexico (26.68 %)
Jawla paste shrimp	*Acetes indicus*	37,085.69	Indonesia (100 %)
Atlantic seabob	*Xiphopenaeus kroyeri*	31,259.21	Brazil (41.91 %), Guyana 929.32 %), Suriname (21.75 %)

Ocean shrimp	*Pandalus jordani*	30,997.00	USA (100 %)
Blue shrimp	*Penaeus stylirostris*	29,685.83	Mexico (94.61 %)
Deep-water rose shrimp	*Parapenaeus longirostris*	29,263.64	Italy (23.92 %), Greece (11.77 %), Tunisia (10.80 %)
Yellowleg shrimp	*Penaeus californiensis*	27,373.63	Mexico (95.89 %)
Common shrimp	*Crangon crangon*	24,892.73	Netherlands (54.98 %), Germany (35.13 %)
Southern pink shrimp	*Penaeus notialis*	23,505.29	Nigeria (76.45 %), Senegal (16.46%)
Coarse shrimp	*Trachypenaeus granulosus*	21,346.14	Indonesia (100 %)
Titi shrimp	*Protrachypene precipua*	10,650.15	Ecuador (97.56 %)
Pacific shrimps nei	Pandalus spp, Pandalopsis spp	9,697.00	USA (100 %)
Parapenaeopsis shrimps nei	*Parapenaeopsis spp*	9,659.00	Pakistan (100 %)
Redspotted shrimp	*Penaeus brasiliensis*	9,000.00	Brazil (100 %)
Pandalus shrimps nei	Pandalus spp	8,996.36	Canada (100 %)
Green tiger prawn	*Penaeus semisulcatus*	8,994.48	Saudi Arabia (33.92 %), Bahrain (31.24 %), Iraq (14.14 %)
Northern pink shrimp	*Penaeus duorarum*	8,523.83	USA (65.57 %), Mexico (31.07 %)
Indian white prawn	*Penaeus indicus*	7,355.94	Indonesia (45.90 %), Bangladesh (35.28 %), India (16.31 %)
Speckled shrimp	*Metapenaeus monoceros*	6,524.00	Bangladesh (77.85 %), Tunisia (22.15 %)
Caramote prawn	*Penaeus kerathurus*	6,208.45	Tunisia (65.56 %), Italy (14.66%), Libya (8.30 %)
Southern white shrimp	*Penaeus schmitti*	5,327.32	Brazil (65.70 %), Venezuela (Boliv Rep of) (34.30 %)
Tsivakihini paste shrimp	*Acetes erythraeus*	5,000.00	Mozambique (100 %)
Rainbow shrimp	*Parapenaeopsis sculptilis*	4,477.36	Indonesia (100 %)
Chilean nylon shrimp	*Heterocarpus reedi*	4,462.00	Chile (100 %)
Argentine red shrimp	*Pleoticus muelleri*	2,865.53	Brazil (97.91 %)
Western school shrimp	*Metapenaeus dalli*	2,859.20	Australia (100 %)
Western king prawn / King prawn	*Penaeus latisulcatus / Melicertus latisulcatus*	2,314.39	Indonesia (80.30 %), Thailand (19.70 %)
Giant red shrimp	*Aristaeomorpha foliacea*	2,296.22	Italy (94.73 %)
Blue and red shrimp	*Aristeus antennatus*	2,251.34	Spain (32.19 %), Italy (31.30 %), Algeria (23.92 %)
Striped red shrimp	*Aristeus varidens*	2,031.29	Spain (99.95 %)
Endeavour shrimp	*Metapenaeus endeavouri*	1,574.20	Australia (54.09 %), Philippines (45.91%)
Coonstripe shrimp	*Pandalus hypsinotus*	1,562.00	Russian Federation (100 %)

Kadal shrimp	*Metapenaeus dobsoni*	1,302.07	Indonesia (100 %)
Shiba shrimp	*Metapenaeus joyneri*	1,,259.40	Korea, Republic of (100 %)
Crystal shrimp	*Penaeus brevirostris*	1186.92	Panama (72.84 %), El Salvador (17.50 %)
Pacific seabobs	*Xiphopenaeus, Trachypenaeus spp*	1,117.32	El Salvador (85.14 %), Panama (7.21 %)
Coral shrimp	*Parapenaeopsis cornuta*	1,025.70	Indonesia (99.90 %)
Jinga shrimp	*Metapenaeus affinis*	1,010.00	Saudi Arabia (100 %)
Eastern school shrimp	*Metapenaeus macleayi*	1,007.05	Australia (100 %)
**Miscellaneous marine crustaceans**			
Squillids nei	Squillidae	220,930.55	China (99.45 %)
Marine crustaceans nei	Crustacea	132,549.38	Bangladesh (34.93 %), Indonesia (27.42 %), Sri Lanka (15.03 %)
Stomatopods nei	Stomatopoda	8,009.29	India (83.67 %), Thailand (16.05 %)
Giant harpiosquillid mantis sh	*Harpiosquilla raphidea*	7,652.50	Indonesia (100 %)
Spottail mantis squillid	*Squilla mantis*	5,225.92	Italy (76.76 %), Spain (20.93 %)
[Calanus finmarchicus]	*Calanus finmarchicus*	1,156.00	Norway (100 %)

Table S6. Total global production of the most important Molluscan shellfish in 2021, including aquaculture and capture production. The species produced in > 1000 tones and their main producers are presented. The data are mined from FAO database (https://www.fao.org/fishery/en/statistics/software/fishstatj).

Common name	Latin name	Tons of live weight	The main producers (% of total production)
**Freshwater molluscs**			
Freshwater molluscs nei	Mollusca	212,045.18	China (76.32 %), Philippines (14.61 %), Korea, Republic of (3.81 %)
Chinese mystery snail	*Cipangopaludina chinensis*	98,420.00	China (100 %)
Chinese pond mussel	*Sinanodonta woodiana*	54,628.00	China (100 %)
Asian clam	*Corbicula fluminea*	26,385.74	China (84.31 %), Taiwan Province of China (15.69 %)
Japanese corbicula	*Corbicula japonica*	9,560.00	Japan (93.51 %), Russian Federation (6.48 %)
**Abalones, winkles, conchs**			
Sea snails	Rapana spp	299,620.00	China (100 %)
Abalones nei	Haliotis spp	245,567.51	China (88.71 %), Korea, Republic of (9.49 %)
Gastropods nei	Gastropoda	40,514.86	Mexico (34.56%), Korea, Republic of (22.10 %), Russian Federation (17.21 %)
Whelk	*Buccinum undatum*	38,276.59	United Kingdom (49.46 %), France (31.29 %), Ireland (15.16 %)
Stromboid conchs ne	*Strombus spp*	32,588.57	Nicaragua (50.36 %), Belize (12.27 %), Turks and Caicos Is. (6.47 %)
Veined rapa whelk	*Rapana venosa*	12,171.68	Ukraine (40.59 %), Romania (22.56 %), Russian Federation (19.04 %)
Horned turban	*Turbo cornutus*	6,852.23	Japan (62.75%), Korea, Republic of (37.25 %)
Pig's snout volute	*Cymbium cymbium*	3,238.00	Mauritania (100 %)
False abalone	*Concholepas concholepas*	2,922.15	Chile (80.52%), Peru (19.48 %)
Volutes nei	*Cymbium spp*	2,869.91	Senegal (99.58 %)
Perlemoen abalone	*Haliotis midae*	2,423.88	South Africa (97.13 %)
Spiral babylon	*Babylonia spirata*	2,368.00	Pakistan (100 %)
Purple dye murex	*Bolinus brandaris*	1,824.42	Italy (84.45%), France (11.23 %)
Changeable nassa	*Tritia mutabilis*	1,365.45	Italy (85.12%), France (14.57 %)
Red abalone	*Haliotis rufescens*	1,320.95	Chile (84.94%), USA (11.66 %), Mexico (3.40 %)
Turbans nei	Turbo spp	1,068.00	USA (100 %)

**Oysters**			
Cupped oysters nei	Crassostrea spp	5,858,347.36	China (99.33 %)
Pacific cupped oyster	*Crassostrea gigas*	652,011.94	Korea, Republic of (50.60%), Japan (24.29 %), France (13.06 %)
American cupped oyster	*Crassostrea virginica*	226,485.41	USA (75.07 %), Mexico (20.51 %), Canada (4.42 %)
Slipper cupped oyster	*Crassostrea Iredalei*	40,799.75	Philippines (100 %)
Flat and cupped oysters nei	Ostreidae	8,051.32	Australia (100 %)
Cortez oyster	*Crassostrea corteziensis*	5,155.78	Mexico (100 %)
European flat oyster	*Ostrea edulis*	4,823.59	Norway (44.85 %), France (29.65 %), Ireland (9.13 %)
Indian backwater oyster	*Crassostrea madrasensis*	4,200.00	India (100 %)
Chilean oyster	*Ostrea chilensis*	1,290.15	New Zealand (60.24%), Chile (39.76 %)
Mangrove cupped oyster	*Crassostrea rhizophorae*	1,049.68	Cuba (98.79%)
**Mussels**			
Sea mussels nei	Mytilidae	1,062,460.81	China (78.07 %), Spain (19.13 %)
Chilean mussel	*Mytilus chilensis*	425,833.00	Chile (100 %)
Blue mussel	*Mytilus edulis*	188,669.96	France (29.23 %), Denmark (17.64 %), Netherlands (Kingdom of the) (17.41 %)
Green mussel	*Perna viridis*	117,603.10	Thailand (44.28 %), Indonesia (21.18 %), Philippines (20.18 %)
New Zealand mussel	*Perna canaliculus*	98,151.73	New Zealand (100 %)
Mediterranean mussel	*Mytilus galloprovincialis*	97,465.55	Italy (64.16%), Greece (14.05 %), France (6.71 %)
Korean mussel	*Mytilus unguiculatus*	64,065.42	Korea, Republic of (100 %)
South American rock mussel	*Perna perna*	9,305.71	Brazil (99.49%)
Cholga mussel	*Aulacomya ater*	7,515.69	Chile (82.37 %), Peru (17.60 %)
Choro mussel	*Choromytilus chorus*	2,550.00	Chile (100 %)
Australian mussel	*Mytilus planulatus*	2,424.70	Australia (100 %)
**Scallops, pectens**			
Scallops nei	Pectinidae	1,834,531.33	China (99.75 %)
Yesso scallop	*Patinopecten yessoensis*	554,409.08	Japan (93.88 %), Russian Federation (5.10 %)
American sea scallop	*Placopecten magellanicus*	220,861.24	USA (73.99 %), Canada (26.%)
Peruvian calico scallop	*Argopecten purpuratus*	112,904.15	Peru (96.55 %)
Great Atlantic scallop	*Pecten maximus*	72,447.55	France (54.20 %), United Kingdom (38.62 %), Ireland (3.79 %)

Patagonian scallop	*Zygochlamys patagonica*	44,883.86	Argentina (99.86 %)
Pen shells nei	*Atrina spp*	12,145.75	China (99.99 %)
Queen scallop	*Aequipecten opercularis*	9,898.02	Faroe Islands (42.67 %), France (25.70 %), United Kingdom (20.63 %)
Southern Australia scallop	*Pecten fumatus*	2,807.60	Australia (100 %)
Pacific calico scallop	*Argopecten ventricosus*	1,947.73	Mexico (100 %)
Asian moon scallop	*Amusium pleuronectes*	1,192.99	Indonesia (100 %)
**Clams, cockles, arkshells**			
Japanese carpet shell	*Ruditapes philippinarum*	4,358,635.76	China (98.15 %)
Constricted tagelus	*Sinonovacula constricta*	859,651.00	China (100 %)
Blood cockle	*Tegillarca granosa*	496,118.64	China (68.61%), Indonesia (18.74 %), Thailand (7.47 %)
Clams, etc. nei	Bivalvia	124,038.51	Korea, Dem. People's Rep (49.98 %), Japan (18.87 %), Korea, Republic of (7.69 %)
Ocean quahog	*Arctica islandica*	85,521.00	USA (99.99 %)
Atlantic surf clam	*Spisula solidissima*	60,675.00	USA (99.43 %)
Japanese hard clam	*Meretrix lusoria*	53,066.95	Taiwan Province of China (99.84 %)
Striped venus	*Chamelea gallina*	37,994.38	Italy (52.26 %), Spain (3.14 %)
Stimpson's surf clam	*Spisula polynyma*	35,650.60	Canada (100 %)
Northern quahog(=Hard clam	*Mercenaria mercenaria*	32,996.32	USA (96.60 %)
Common edible cockle	*Cerastoderma edule*	24,978.05	Denmark (38.70 %), United Kingdom (29.90 %), Portugal (12.88 %)
Ark clams nei	Arca spp	22,169.49	Venezuela (96.13%)
Venus clams nei	Veneridae	18,465.62	Mexica (99.18 %)
Solid surf clam	*Spisula solida*	15,327.21	Netherlands (72.56 %), Portugal (9.58 %), Denmark (9.19 %)
Antique ark	*Anadara antiquata*	14,130.74	Indonesia (100 %)
Taca clam	*Leukoma thaca*	12,765.00	Chile (100 %)
Pod razor shell	*Ensis ensis*	9,048.17	Netherlands (99.89 %)
Sand gaper	*Mya arenaria*	7,591.76	USA (71.06 %), Canada (18.96 %), Bulgaria (9.99 %)
Short neck clams nei	Paphia spp	7,239.28	Thailand (99.99 %)
Grooved carpet shell	*Ruditapes decussatus*	7,185.57	Portugal (71.35 %), Tunisia (24.35 %)
Half-crenated ark	*Scapharca subcrenata*	4,506.93	Korea, Republic of (100 %)

Common European bittersweet	*Glycymeris glycymeris*	4,399.49	France (98.17 %)
Pacific geoduck	*Panopea generosa*	3,588.69	USA (64.12 %), Canada (35.88 %)
Cockles nei	Cardiidae	3,172.24	Canada (85.70 %), Korea, Republic of (14.10 %)
Smooth callista	*Callista chione*	1,699.71	Italy (67.43 %), France 913.27 %), Portugal (9.63 %)
Taquilla clams	Mulinia spp	1,618.00	Chile (100 %)
Undulate venus	*Paratapes undulatus*	1,494.80	Indonesia (100 %)
Inflated ark	*Scapharca broughtonii*	1,409.47	Korea, Republic of (100 %)
Triangular tivela	*Tivela mactroides*	1,355.00	Brazil (100 %)
Butter clam	*Saxidomus giganteus*	1,280.39	Canada (95.28%), USA (4.72 %)
Macha clam	*Mesodesma donacium*	1,273.00	Chile (100 %)
Gay's little venus	*Tawera gayi*	1,258.00	Chile (100 %)
Stutchbury's venus	*Chione stutchburyi*	1,208.31	New Zealand (100 %)
Solen razor clams nei	Solen spp	1,171.54	United Kingdom (71.22 %), Ireland (26.02 %)
Dombey's tagelus	*Tagelus dombeii*	1,065.97	Peru (100 %)
Donax clams	Donax spp	961.36	Portugal (51.03 %), Italy (47.62 %)
**Squids, cuttlefishes, octopuses**			
Jumbo flying squid	*Dosidicus gigas*	1,004,277.76	Peru (51.55 %), China (42.02 %), Chile (5.33 %)
Various squids nei	Loliginidae, Ommastrephidae	542,466.49	China (62.51 %), India (13.93 %), Morocco (5.40 %)
Argentine shortfin squid	*Illex argentinus*	447,091.58	Taiwan Province of China (32.61 %), China (31.25 %), Argentina (29.57 %)
Cephalopods nei	Cephalopoda	423,596.21	Viet Nam (84.87 %), China (10.51 %), Magadascar (2.48 %)
Common squids nei	Loligo spp	332,201.68	Indonesia (61.46 %), Thailand (19.60%), Philippines (13.98 %)
Cuttlefish, bobtail squids nei	Sepiidae, Sepiolidae	326,921.97	China (38.60 %), India (20.84 %), Morocco (9.43 %)
Octopuses, etc. nei	Octopodidae	314,250.12	China (33.83 %), Morocco (20.22 %), Mauritania (8.68 %)
Japanese flying squid	*Todarodes pacificus*	99,540.20	Korea, Republic of (61.13 %), Japan (31.44 %)
Patagonian squid	*Dorytheutis (Amerigo) gahi*	97,624.89	Falkland Is.(Malvinas) (97.29 %)
Schoolmaster gonate squid	Berryteuthis magister	76,444.00	Russian Federation (100 %)
Opalescent inshore squid	*Loligo opalescens*	57,121.39	USA (85.19 %), Mexico (14.81 %)
Common octopus	*Octopus vulgaris*	36,929.78	Mexico (39.99 %), Portugal (17.83 %), Italy (10.32 %)

Northern shortfin squid	*Illex illecebrosus*	34,169.41	USA (51.82 %), Canada (32.97%), Spain (14.02 %)
Wellington flying squid	*Nototodarus sloanii*	29,832.69	New Zealand (100 %)
Common cuttlefish	*Sepia officinalis*	24,894.40	France (27.76 %), Tunisia (21.85 %), Italy (15.31 %)
Mexican four-eyed octopus	*Octopus maya*	20,722.57	Mexico (100 %)
Pharaoh cuttlefish	*Sepia pharaonis*	18,300.09	Oman (75.87 %), Iran (Islamic Rep. of) (21.12 %)
Longfin squid	*Dorytheutis (Amerigo) pealeii*	10,633.00	USA (100 %)
Bigfin reef squid	*Sepioteuthis lessoniana*	8,759.72	Thailand (100 %)
Neon flying squid	*Ommastrephes bartramii*	4,300.11	Japan (100 %)
Broadtail shortfin squid	*Illex coindetii*	3,572.27	France (36.58 %), Spain (30.40 %), Italy (25.66 %)
Inshore squids nei	Loliginidae	3,112.69	France (99.20 %)
European squid	*Loligo vulgaris*	2,215.80	Netherlands (38.04 %), Italy (21.65 %), Spain (28.10 %)
Cape Hope squid	*Loligo reynaudii*	2,098.36	South Africa (99.83 %)
European flying squid	*Todarodes sagittatus*	2,059.47	Spain (32.70 %), Italy (24.74 %), Ireland (12.87 %)
Horned octopus	*Eledone cirrhosa*	1,908.74	Italy (47.71 %), Spain (41.30 %), Portugal (10.93 %)
Horned and musky octopuses	Eledone spp	1,324.43	Tunisia (49.46 %), Spain (29.20 %), Croatia (13.79 %)
Musky octopus	*Eledone moschata*	1,124.74	Italy (90.90 %)
**Miscellaneous marine molluscs**			
Marine molluscs nei	Mollusca	1,680,333.69	China (66.78 %), Viet Nam (24.07 %), India (6.27 %)

Table S7. Top 20 countries with the highest Cephalopoda export in 2021. The data are mined from FAOSTAT database (https://www.fao.org/faostat/en/)

	**Cephalopoda export**	**Kilotons**	**% of global export**
1	**China, mainland**	628.29	**23.93**
2	Peru	503.15	19.17
3	Spain	214.05	8.15
4	India	189.09	7.20
5	Indonesia	149.37	5.69
6	Viet Nam	91.72	3.49
7	Morocco	90.48	3.45
8	Argentina	84.42	3.22
9	United States of America	55.79	2.13
10	Thailand	53.71	2.05
11	Mauritania	48.65	1.85
12	China, Taiwan Province of	45.75	1.74
13	Chile	42.9	1.63
14	New Zealand	39.53	1.51
15	Malaysia	39.13	1.49
16	Russian Federation	38.62	1.47
17	Myanmar	34.67	1.32
18	Portugal	27.62	1.05
19	France	18.78	0.72
20	Ecuador	18.54	0.71
	Global export	2625.12	
	Top 20 contries export (% of global export)		**91.97**

Table S8. Top 20 countries with the highest Crustaceas export in 2021. The data are mined from FAOSTAT database (https://www.fao.org/faostat/en/).

	Crustacea export	Kilotons	% of global export
1	**India**	729.67	**14.04**
2	Ecuador	697.14	13.42
3	China, mainland	673.75	12.97
4	Viet Nam	522.6	10.06
5	Indonesia	429.58	8.27
6	Thailand	308.13	5.93
7	Canada	277.06	5.33
8	Netherlands	177.81	3.42
9	Argentina	167.08	3.22
10	Denmark	157.51	3.03
11	Myanmar	155.12	2.99
12	United Arab Emirates	113.21	2.18
13	United States of America	109.56	2.11
14	Malaysia	104.02	2.00
15	Republic of Korea	97.55	1.88
16	Russian Federation	97.18	1.87
17	Belgium	93.13	1.79
18	United Kingdom of Great Britain and Northern Ireland	76.65	1.48
19	Norway	69.96	1.35
20	Spain	66.22	1.27
	Global export	5195.67	
	Top 20 contries export (% of global export)		**98.60**

Table S9. Top 20 countries with the highest Mollusca ex. Cephalopoda export in 2021. The data are mined from FAOSTAT database (https://www.fao.org/faostat/en/).

	**Mollusca ex. Cephalopoda export**	**Kilotons**	**% of global export**
**1**	**China, mainland**	**1205.39**	**37.59**
2	Japan	282.68	8.81
3	Chile	240.42	7.50
4	Spain	174.6	5.44
5	Thailand	130.23	4.06
6	Viet Nam	118.08	3.68
7	Netherlands	97.02	3.03
8	New Zealand	96.86	3.02
9	Canada	91.37	2.85
10	Republic of Korea	69.27	2.16
11	United Kingdom of Great Britain and Northern Ireland	68.37	2.13
12	United States of America	67.84	2.12
13	France	58.46	1.82
14	Denmark	58.45	1.82
15	Italy	44.54	1.39
16	Germany	41.98	1.31
17	Indonesia	40.48	1.26
18	Portugal	39.11	1.22
19	Ireland	33.85	1.06
20	Russian Federation	30.98	0.97
	Global export	3206.97	
	Top 20 contries export (% of global export)		**97.20**

Table S10. Top 20 countries with the highest Cephalopoda import in 2021. The data are mined from FAOSTAT database (https://www.fao.org/faostat/en/).

	**Cephalopoda import**	**Kilotons**	**% of global import**
**1**	**China, mainland**	**428.91**	**16.96**
2	Spain	348.42	13.78
3	Republic of Korea	251.8	9.96
4	Japan	241.31	9.54
5	Italy	218.58	8.64
6	Thailand	200.76	7.94
7	United States of America	169.44	6.70
8	Portugal	62	2.45
9	France	58.93	2.33
10	China, Taiwan Province of	49.62	1.96
11	Malaysia	44.41	1.76
12	Philippines	35.62	1.41
13	Russian Federation	33.5	1.32
14	Greece	32.97	1.30
15	Viet Nam	32.77	1.30
16	Australia	27.64	1.09
17	China, Hong Kong SAR	26.19	1.04
18	Germany	25.07	0.99
19	Netherlands (Kingdom of the)	16.36	0.65
20	United Kingdom of Great Britain and Northern Ireland	16.35	0.65
	Global import	2528.79	
	Top 20 contries import (% of global import)		**91.77**

Table S11. Top 20 countries with the highest Crustaceans import in 2021. The data are mined from FAOSTAT database (https://www.fao.org/faostat/en/).

	**Crustacea import**	**Kilotons**	**% of global import**
**1**	**United States of America**	**1895.03**	**25.66**
2	China, mainland	1347.93	18.25
3	Japan	892.96	12.09
4	Canada	251.52	3.41
5	France	250.06	3.39
6	United Kingdom of Great Britain and Northern Ireland	215.8	2.92
7	Spain	210.29	2.85
8	Republic of Korea	199.45	2.70
9	Denmark	184.17	2.49
10	Netherlands (Kingdom of the)	169.02	2.29
11	Italy	116.97	1.58
12	Germany	110.35	1.49
13	Belgium	110.01	1.49
14	China, Hong Kong SAR	107.19	1.45
15	United Arab Emirates	90.21	1.22
16	Sweden	83.95	1.14
17	Thailand	80.74	1.09
18	China, Taiwan Province of	80.35	1.09
19	Russian Federation	69.68	0.94
20	Australia	68.84	0.93
	Global import	7384.89	
	Top 20 contries import (% of global import)		**88.49**

Table S12. Top 20 countries with the highest Mollusca ex. Cephalopoda import in 2021. The data are mined from FAOSTAT database (https://www.fao.org/faostat/en/).

	**Mollusca ex. Cephalopoda import**	**Kilotons**	**% of global import**
**1**	**United States of America**	**420.64**	**13.64**
2	China, mainland	402.85	13.06
3	Japan	271.13	8.79
4	Italy	228.97	7.42
5	France	206.92	6.71
6	Republic of Korea	181.37	5.88
7	Spain	172.26	5.58
8	China, Hong Kong SAR	125.35	4.06
9	Portugal	109.83	3.56
10	Thailand	95.96	3.11
11	China, Taiwan Province of	91.03	2.95
12	Russian Federation	86.04	2.79
13	Netherlands	68.84	2.23
14	Canada	57.76	1.87
15	Belgium	51.08	1.66
16	Australia	50.27	1.63
17	Malaysia	47.6	1.54
18	Germany	46.24	1.50
19	Cambodia	45.13	1.46
20	Ukraine	39.72	1.29
	Global import	3084.58	
	Top 20 contries import (% of global import)		**90.74**

Section S2.2. Shellfish supply per capita

Oceania produces 100 times lower quantities of shellfish than Asia, but has a similar supply level per capita, which is due to the 100 times higher population in Asia in comparison to Oceania. Africa is the lowest producer/exporter/importer and the lowest consumer of shellfish. Europe has a high supply of shellfish per capita in relation to its low production, mainly relying on import. The supply quantity of Crustaceans per capita is the highest in Americas and Oceania; Asia has the highest supply per capita is for Mollusc ex Cephalopodes (Figure 4), while Europe has a

balanced supply per capita for Crustacea and Mollusc. It also has the highest supply per capita for Cephalopodes.

The global fish and seafood market is likely to grow at a substantial rate during the forecast period, between 2023 and 2030. Production, supply per capita and consumption may rapidly change in the coming years. Increasing population in developing, newly industrialized, and developed countries are now able to afford even highly prized seafood, such as lobsters, prawns, and fresh fish. This development emerged from increasingly globalized trading systems, in which fresh goods, alive or chilled, can be shipped at virtually any time to virtually anywhere on the planet. Increases in seafood consumption rates, trade, and the number of trade routes can largely be explained by concomitant increases in individual wealth (Watson et al., 2016)

Table S13. Top 20 countries with the highest Crustacea, Mollusca (excluded Cephalopoda) and Cephalopoda supply quantity per capita per year in 2021. The data are mined from FAO database. Asian countries are bolded.

	**Crustacea**		**Mollusca (excluded Cephalopoda)**		**Cephalopoda**	
	**Country**	**kg/capita/yr**	**Country**	**kg/capita/yr**	**Country**	**kg/capita/yr**
1	**China, Macao SAR**	15.29	**China, Macao SAR**	18.82	**Republic of Korea**	6.66
2	Iceland	13.28	**China, Hong Kong SAR**	14.22	Seychelles	4.58
3	**China, Hong Kong SAR**	12.64	**China, mainland**	10.09	Portugal	4.19
4	Guyana	12.41	**Republic of Korea**	9.83	Cyprus	3.99
5	Norway	10.19	Belize	7.99	Italy	3.78
6	Bahamas	9.3	Portugal	7.91	Greece	3.43
7	Sweden	7.09	Antigua and Barbuda	6.66	**Thailand**	3.43
8	Denmark	6.74	**China, Taiwan Province of**	6.64	Spain	3.34
9	Antigua and Barbuda	6.6	**Japan**	5.53	**China, Hong Kong SAR**	3.21
10	**Japan**	6.35	France	5.49	**Viet Nam**	3.21
11	United States of America	6.06	Italy	5.06	Croatia	2.95
12	**China, mainland**	6	Spain	4.98	**Japan**	2.77
13	Seychelles	5.51	Luxembourg	4.5	**China, Macao SAR**	2.69
14	**Viet Nam**	5.25	**Cambodia**	4.01	Malaysia	2.26
15	Luxembourg	4.82	Saint Kitts and Nevis	3.91	**China, Taiwan Province of**	2.04
16	**Maldives**	4.11	Saint Lucia	3.72	Slovenia	1.96
17	**Indonesia**	3.96	Malta	3.62	New Zealand	1.71
18	Portugal	3.84	Fiji	3.4	Mauritius	1.68
19	**China, Taiwan Province of**	3.83	Canada	3.2	Panama	1.61
20	**Malaysia**	3.78	French Polynesia	2.99	Montenegro	1.45

Section S3. Allergens of shellfish

Section S3.1. Shellfish allergens registered in WHO/IUIS

Table S14. Crustacean food allergens from the database of the World Health Organization and International Union of Immunological Societies (WHO/IUIS) Allergen Nomenclature Sub-committee (https://allergen.org/index.php)

Species	GenBank Nucleotide	GenBank Protein	UniProt	Allergen	Biochemical name	MW
Archaeopotamobius sibiriensis (**Crayfish**), Class: Malacostraca, Order: Decapoda, **freshwater**	AJ439988	CAD29196	Q8T5G9	**Arc s 8.0101**	Triosephosphate isomerase	~28 kDa
Artemia franciscana (**Brine shrimp**), Class: Branchiopoda, Order: Anostraca, **inland saltwater and marine**	EF660909	ABS19977	A7L499	**Art fr 5.0101**	Myosin, light chain 1	~17.5 kDa
Callinectes bellicosus (**Warrior swimming brown crab**), Class: Malacostraca, Order: Decapoda, **marine**	MW602301	n.a.	A0A976YI25	**Cal b 2.0101**	Arginine kinase	40 kDa
Charybdis feriatus (**Crab**), Class: Malacostraca, Order: Decapoda, **marine**	AF061783	AAF35431	Q9N2R3	**Cha f 1.0101**	Tropomyosin	34 kDa
Charybdis feriatus (**Crab**), Class: Malacostraca, Order: Decapoda, **marine**	PP526952	WXT82172	n.a.	**Cha f 10.0101**	Fructose bisphosphate aldolase	41 kDa
Crangon crangon (**North Sea shrimp**), Class: Malacostraca, Order: Decapoda, **marine**	FJ457621	ACR43473	D7F1J4	**Cra c 1.0101**	Tropomyosin	~38 kDa
	FJ457622	ACR43474	D7F1J5	**Cra c 2.0101**	Arginine kinase	~45 kDa
	FJ462737	ACR43475	D7F1P9	**Cra c 4.0101**	Sarcoplasmic calcium-binding protein	~25 kDa
	FJ462739	ACR43477	D7F1Q1	**Cra c 5.0101**	Myosin, light chain 1	~17.5 kDa
	FJ462740	ACR43478	D7F1Q2	**Cra c 6.0101**	Troponin C	~21 kDa
	FJ462738	ACR43476	D7F1Q0	**Cra c 8.0101**	Triosephosphate isomerase	~28 kDa
Eriocheir sinensis (**Chinese mitten crab**), Class: Malacostraca, Order: Decapoda, **freshwater and marine**	AY185917	AAO73305	Q5QKR2	**Eri s 2.0101**	ovary development-related protein	28.2 kDa

Exopalaemon modestus (**White legged fresh water shrimp**), Class: Malacostraca, Order: Decapoda, **freshwater**	n.a.	n.a.	Submitted	**Exo m 1.0101**	Tropomyosin	38 kDa
Homarus americanus (**American lobster**), Class: Malacostraca, Order: Decapoda, **marine**	AF034953	AAC48287	O44119-1	**Hom a 1.0101**	Tropomyosin	34 kDa
	AF034954	AAC48288	O44119-2	***Hom a 1.0102***	Tropomyosin	34 kDa
	EH115965	KAG71677 62	n.a.	**Hom a 3.0101**	Myosin light chain 2	~23 kDa
	n.a.	P29291	P29291	**Hom a 6.0101**	Troponin C	~20 kDa
Litopenaeus vannamei (**Whiteleg shrimp**), Class: Malacostraca, Order: Decapoda, **marine**	EU410072	ACB38288	B4YAH6	**Lit v 1.0101**	Tropomyosin	36 kDa
	DQ975203	ABI98020	Q004B5	**Lit v 2.0101**	Arginine kinase	40 kDa
	EU449515	ACC76803	B7SNI3	**Lit v 3.0101**	Myosin, light chain 2	20 kDa
	FJ184279	ACM89179	C7A639	**Lit v 4.0101**	Sarcoplasmic calcium-binding protein	20 kDa
	HM535967	ADK66280	E2IH93	**Lit v 13.0101**	Fatty Acid Binding Protein; FABP	15 kDa
Macrobrachium rosenbergii (**Giant freshwater prawn**), Class: Malacostraca, Order: Decapoda, **freshwater**	GU369816	ADC55380	D3XNR9	**Mac r 1.0101**	Tropomyosin	37 kDa
	HQ191218	ADN88091	E2JE77	**Mac r 2.0101**	Arginine kinase	40 kDa
Melicertus latisulcatus (**King prawn**), Class: Malacostraca, Order: Decapoda, **marine**	JX171685	AGF86397	M4M2H6	**Mel l 1.0101**	Tropomyosin	38 kDa
Metapenaeus ensis (**Shrimp**), Class: Malacostraca, Order: Decapoda, **marine**	U08008	AAA60330	Q25456	**Met e 1.01*01***	Tropomyosin	34 kDa
Pandalus borealis (**Northern shrimp**), Class: Malacostraca, Order: Decapoda, **marine**	FR728681	CBY17558	E5BBS3	**Pan b 1.0101**	Tropomyosin	37 kDa
Panulirus stimpsoni (**Spiny lobster**), Class: Malacostraca, Order: Decapoda, **marine**	AF030063	AAC38996	O61379	**Pan s 1.0101**	Tropomyosin	34 kDa
Paralithodes camtschaticus ***(Red king crab)***	PP541528	WYC14267	n.a.	**Para c 11.0101**	Mitochondrial malate dehydrogenase	39 kDa
Penaeus aztecus (**Brown shrimp**), Class: Malacostraca, Class: Malacostraca, Order: Decapoda, **marine**	DQ151457	AAZ76743	Q3Y8M6	**Pen a 1.0101**	Tropomyosin	36 kDa

Penaeus indicus (**Shrimp**), Class: Malacostraca, Order: Decapoda, **marine**	n.a.	n.a.	n.a.	**Pen i 1.0101**	Tropomyosin	34 kDa
Penaeus monodon (**Black tiger shrimp**), Class: Malacostraca, Order: Decapoda, **marine**	AY827100, AB270629, HM486525	AAX37288, BAF47262, ADM34184	A1KYZ2, E1A682	**Pen m 1.0101**	Tropomyosin	38 kDa
	AF479772	AAO15713	Q8I9P7	**Pen m 2.0101**	Arginine kinase	40 kDa
	HM034314, HM486526	ADV17342, ADM34185	E1A683	**Pen m 3.0101**	Myosin light chain 2	20 kDa
	HM034315	ADV17343	E7CGC4	**Pen m 4.0101**	Sarcoplasmic calcium binding protein	20 kDa
	HM034316	ADV17344	E7CGC5	**Pen m 6.0101**	Troponin C	16.8 kDa
	JF357966.1	AEB77775.1	G1AP69	***Pen m 7.0101***	Hemocyanin	76 kDa
	GQ328754	ADG86240	F8QN77	**Pen m 8.0101**	Triosephosphate isomerase	27 kDa
	JN572542	AEP84100	Q1KS35	**Pen m 13.0101**	Cytoplasmic Fatty Acid Binding Protein	20 kDa
	OM156460	URW11955	n.a.	**Pen m 14.0101**	Glycogen phosphorylase-like protein	95 kDa
Pontastacus leptodactylus (Narrow-clawed crayfish), Class: Malacostraca, Order: Decapoda, fresh and brackish waters	n.a.	P05946	P05946	**Pon l 4.0101**	Sarcoplasmic calcium-binding protein	~24 kDa
	n.a.	P05547	P05547	**Pon l 7.0101**	Troponin I	~30 kDa
Portunus pelagicus (**Blue swimmer crab**), Class: Malacostraca, Order: Decapoda, **marine**	JX874982	AGE44125	M1H607	**Por p 1.0101**	Tropomyosin	39 kDa
Procambarus clarkii (**Red swamp crayfish**), Class: Malacostraca, Order: Decapoda, **freshwater**	FJ769183	ACN87223	C0LU07	**Pro c 1.0101**	Tropomyosin	36 kDa
	JN828651	ACN87223	H6VGI2	**Pro c 2.0101**	Arginine kinase	40 kDa
	JX173714	AFP95338	J7HCX7	**Pro c 5.0101**	Myosin light chain 1	18 kDa
	HQ414580	AEB54655	F5A6E9	**Pro c 8.0101**	Triosephosphate isomerase	28 kDa
Scylla paramamosain (**Mud crab**), Class: Malacostraca, Order: Decapoda, **marine**	EF672351.1 *Scylla serrata*	ABS12233.1	A7L5V2* (TPM *Scylla serrata*)	**Scy p 1.0101**	Tropomyosin	38 kDa

Scylla serrata is a different taxon – IUIS under *Scylla paramamosain* TPM links *Scylla serrata* TPM		*Scylla serrata*	A0A6C0N3G5 (suggested correction of entry)			
	JN828652	AFA45340	H6VGI3	**Scy p 2.0101**	Arginine kinase	40 kDa
	MK749844.1	QDH76468.1	A0A514C9K9	**Scy p 3.0101**	Myosin light chain	18 kDa
	JQ860424	AFJ80778	I2DDG2	**Scy p 4.0101**	Sarcoplasmic calcium binding protein	20 kDa
	KX083344	APP94292	A0A1L5YRA2	**Scy p 8.0101**	Triosephosphate isomerase	28 kDa
	MK747241	QFI57017	n.a.	**Scy p 9.0101**	Filamin C	90 kDa

Table S15. Molluscan food allergens from the database of the World Health Organization and International Union of Immunological Societies (WHO/IUIS) Allergen Nomenclature Sub-committee

Species	GenBank Nucleotide	GenBank Protein	UniProt	Allergen	Biochemical name	MW
Crassostrea angulata (**Portuguese oyster**), Class: Bivalvia, Order: Ostreida, **marine**	ON595539	UST29548	n.a.	**Cra a 1.0101**	Tropomyosin	38 kDa
	MW864167	QZP44316.1	n.a.	**Cra a 2.0101**	Arginine kinase	38 kDa to 41 kDa
	MN956521	QIJ32297.1	A0A6G7MAZ4	**Cra a 4.0101**	Sarcoplasmic calcium binding protein	20 kDa
Crassostrea gigas (**Pacific oyster**), Class: Bivalvia, Order: Ostreida, **marine**	KY549366.1	ARX70262.1	n.a.	**Cra g 1.0101**	Tropomyosin	38 kDa
	AB444943.1	BAH10152.1	n.a.	***Cra g 1.0102***	Tropomyosin	38 kDa
Haliotis laevigata x Haliotis rubra (**Jade tiger abalone**), Class: Gastropoda, Order: Lepetellida, **marine**	KX961689	APG42675	A0A1L3HS57	**Hal l 1.0101**	Tropomyosin	33.4 kDa
Haliotis midae (**Perlemoen abalone**), Class: Gastropoda, Order: Lepetellida, **marine**	n.a.	n.a.	n.a.	**Hal m 1.0101**	n.a.	49 kDa
Helix aspersa [Cornu aspersum] (**Brown garden snail**), Class: Gastropoda, Order: Stylommatophora, **terrestrial**	Y14855	CAB3804	**O97192**	**Hel as 1.0101**	Tropomyosin	36 kDa
Rapana venosa (**Veined rapa whelk**), Class: Gastropoda, Order: Neogastropoda, **marine**	MN784956.1	QPB41107	n.a.	**Rap v 2.0101**	Paramyosin	99 kDa
Saccostrea glomerata (**Sydney rock oyster**), Class: Bivalvia, Order: Ostreida, **marine**	MF996471	n.a.	A0A2L1FDX2.	**Sac g 1.0101**	Tropomyosin	38 kDa
Todarodes pacificus (**Japanese flying squid**), Class: Cephalopoda, Order: Oegopsida, **marine**	n.a.	n.a.	n.a.	**Tod p 1.0101**	Tropomyosin	38 kDa

Section S3.2. Availability of recombinant shellfish allergens

Creative BioMart (https://allergen.creativebiomart.net/products/food.html) have the widest palette of shellfish allergens, and currently unavailable are only 6 allergens from Crustacea (Pen m 7, Pen m 14, Para c 11, Mac r 2, Lit v 13 and Cha f 10) and 2 allergens from Mollusca (Cra a 1 and Cra a 2). SinoBiological offers recombinant Crustacean tropomyosins (Met e 1, Pen a 1, Scy p 1, Scy p2, Sci p 3, Scy p 4), but none of Molluscan allergens (https://www.sinobiological.com). MyBioSource (https://www.mybiosource.com/) manufactures 13 recombinant Crustacean (Pen a 1, Pen m 1, Pen m 2, Pen m 3, Pen m 4, Pen m 7, Cha f 1, Hom a 1, Hom a 6, Met e 1, Pan b 1, Pan s 1, Pon l 7) and none of Mollusacan allergens. RayBiotech (https://www.raybiotech.com) have only Pen a 1, Cha f 1 and Met e 1 shellfish allergens. In Causabio (https://www.cusabio.com/) shellfish allergen palette there are 16 Crustacean (Cha f 1, Pen m 1, Pen m 2, Pen m 3, Pen m 4, Pen m 7, Pan b 1, Pan s 1, Hel as 1, Hom a 1, Hom a 6, Bomb m 1, Met e 1, Eri s 2, Cal b 2, Pon l 7) and one Molluscan (Tod p 1) allergen. This shows that all TPMs registered in WHO/IUIS, except Cra a 1, are commercially available.

In addition to shellfish allergens registered in WHO/IUIS, several manufacturers produce also 7 allergens (6 Molluscan and 1 Crustacean) with documented IgE reactivity, but not yet registered in WHO/IUIS. Moreover, these manufacturers offer several recombinant shellfish proteins for which IgE reactivity was not documented yet but are potential shellfish allergens as they are types of proteins known as allergens in shellfish (such as AK, SCBP, troponin C, hemocyanin, actin). Besides, there are many companies offering customized protein expression. Therefore, if complete allergen sequence is known, recombinant allergen could be available. For some shellfish allergens nonregistered in WHO/IUIS (Figure 6), but with demonstrated IgE reactivity, complete gene and protein sequences are available (In detail data available at https://cherry.chem.bg.ac.rs/handle/123456789/7052).

Section S4. Diagnostic and therapy tools for shellfish allergies

Section S4.1. Shellfish allergens within skin prick test diagnostic tools

Table S16. Commercially available shellfish allergen extracts for SPT and *in vitro* allergy diagnostics from several producers: ALK-Abelló (https://www.alk.net/us/sites/www.alk.us/files/2023_alk_product_catalog.pdf), STALLERGENS GREER (https://www.stagrallergy.com/wp-content/uploads/2020/07/SCIT2020_Catalog40p_062920_SP.pdf), DST Diagnostische Systeme & Technologien GmbH (https://www.dst-diagnostic.com/allergen_extracts/), Labor Dr. Weyers (https://allergenextrakt.de/wp-content/uploads/2012/05/KatalogAllergene.pdf), HollisterStier Allergy (https://www.hsallergy.com/allergy-extracts/food-extracts/) and Beijing Macro-Union Pharmaceutical Limited Corporation (http://www.callergen.com/en/index.php/Home/Index/productnw/id/431).

	**Shellfish extracts for SPT**				**Shellfish extracts for *in vitro* diagnostics**	
	**ALK-Abelló**	**STALLERGENS GREER**	**HollisterStier Allergy**	**Beijing Macro-Union**	**DST**	**Labor Dr. Weyers**
Shellfish mixed	MISH: Shellfish, Mixed (Crab, Shrimp, Lobster, Oyster)	FO2: Shellfish Mix Equal parts: F10 Clam, Northern Quahog; F12 Crab, Blue; F23 Oyster, Atlantic/Eastern; F32 Scallops, Sea; F34 Shrimp, Brown	GFO2A01 Shellfish Mix (clam, crab, oyster, scallops, shrimp)	n.a.	n.a.	n.a.
**Crustacea**						
Freshwater crustacean	n.a.	n.a.	n.a.	*Eriocheir sinensis* extract (Chinese mitten crab);	F178 *Procambarus clarkii* extract (Crayfish)	n.a.
Crabs, sea-spiders	CRAB: *Paralithodes camtchatica* extract (Crab Meat, King Crab)	F12 *Callinectes sapidus* (Crab, blue)	GF12A01 Crab	*Portunus trituberculatus* (Gazami crab)	F241 *Pandalus borealis* (Crab)	n.a.
Lobsters	LOBS: *Homarus americanus* (Lobster)	F20 *Homarus americanus* (Lobster, American)	GF20A01 Lobster, Maine	n.a.	F71 *Palinurus elephas* (Spiny lobster); F80 *Homarus gammarus* (Lobster)	F71 *Palinurus vulgaris* (Spiny lobster); F80 *Homarus gammarus* (Lobster)

Shrimps, prawns	SHRI: *Crangon crangon* (Shrimp)	F34 *Farfantepenaeus aztecus* (Shrimp, Brown)	GF34A01 Shrimp	n.a.	F24 *Penaeus monodon (*Shrimp)	F23 *Crangon crangon* (Prawn, North-sea); *Parapenaeus longirostris* (Prawn, Deep-sea)
**Mollusca**						
Oysters	OYST: *Crassostrea virginica* (Oyster)	F23 *Crassostrea virginica* (Oyster, Atlantic/Eastern)	GF23A01 Oyster	n.a.	F177 *Ostrea edulis* (Oyster)	F124 *Ostrea edulis* (Oyster)
Mussels	n.a.	n.a.	n.a.	n.a.	F37 *Mytilus edulis* (Blue mussel)	F37 *Mytilus edulis* (Mussel)
Scallops, pectens	n.a.	F32 *Placopecten magellanicus* (Scallops, sea)	GF32A01 Scallops	n.a.	F338 *Pecten maximus* (Scallop)	n.a.
Clams, cockles, arkshells	CLAM: *Mercenaria mercenaria* (Clam)	F10 *Mercenaria mercenaria* (Clam, Northern Quahog)	GF10A01 Clam	n.a.	F328 *Chlamys varia* (Clam)	F176 *Venus gallina* (Clam)
Squids, cuttlefishes, octopuses	n.a.	n.a.	n.a.	n.a.	F819 *Octopus vulgaris* (Octopus); F176 Loligo spp. (Squid); F108 *Todarodes pacificus* (Pacific squid)	F120 *Sepia officinalis* (Squid/Inkfish)
Abalones, winkles, conchs	n.a.	n.a.	n.a.	n.a.	F935 *Haliotis japonica* (Abalone)	n.a.

Section S4.2. Shellfish allergens within diagnostic tools for measurement of allergen-specific IgE

Table S17. Commercially available extract and allergen componenents within several singleplex and multiplex systems: ImmunoCAP system (Phadia/Thermo Fisher Scientific) (https://www.thermofisher.com/order/catalog/product/14528310?SID=srch-srp-14528310), IMMULITE 1000/2000/XPi Immunoassay Systems (https://marketing.webassets.siemens-healthineers.com/5f4dc04ed9773e8b/4278872702e7/IMMULITE-Allergy-Menu_OUS.pdf?ste_sid=f506de7ef5b84a5c5ebf7652208fbe47), FABER (https://www.caam-allergy.com/en/faber), Alex2 Allergy Explorer (Macroarray Diagnostics, https://ultimavitality.co.uk/wp-content/uploads/2023/01/Alex2-Allergen-List.pdf) and ImmunoCAP ISAC 112 (Thermo Fisher Scientific, https://www.thermofisher.com/diagnostic-education/dam/hcp/documents/Go-Molecular-Book-1-2.pdf). Allergen components are bolded.

	**Singleplex measurement of shellfish specific IgE**			**Multiplex measurement of shellfish specific IgE**	
	**ImmunoCAP**	**IMMULITE 1000/2000/Xpi**	**FABER**	**Alex2 Allergy Explorer**	**ImmunoCAP ISAC 112**
**CRUSTACEA**					
Freshwater crustacean	F320 Crayfish: *Astacus astacus* (Boiled meat and shell from crayfish)	f320 Crayfish extract	n.a.	n.a.	n.a.
Crabs, sea-spiders	F23 Crab: *Cancer pagurus* (Boiled crab meat)	f23 Crab extract	n.a.	f23 Chionoecetes spp extract	n.a.
Lobsters	F80 Lobster: *Homarus gammarus* (Lobster meat); f304 Spiny lobster: Palinurus spp. (Langust meat)	f80 Lobster extract	Homarus americanus extract	f80 *Homarus gammarus* extract	n.a.
Shrimps, prawns	F24 Shrimp: Mixture of *Pandalus borealis, Penaeus monodon, Metapenaeopsis barbata* and *Metapenaeus joyneri* (Boiled, frozen Atlantic shrimp and raw, frozen prawns from the Indo-West-Pacific); f351 **rPen a 1** from *Penaeus aztecus*	f24 Shrimp extract; and **nPen m 1** from *Penaeus monodon*	*Litopenaeus vannamei* extract; **Lit v 1** from *Litopenaeus vannamei*	f517 **nPen m 1,** f545 **nPen m 2,** f552 **nPen m 3** and f524 **rPen m 4** from Penaeus monodon**;** f515 Pandalus borealis extract; f24 mixture of *Penaeus duorarum*, *Pandalus borealis* and *Penaeus setiferus* extract; **rPen a 1** from *Penaeus aztecus*; F529 **rCra c 6** from *Crangon crangon*	**nPen m 1, nPen m 2** and **nPen m 4** from *Penaeus monodon*
**MOLLUSCA**					
Oysters	F290 Oyster *Ostrea edulis* (Fresh whole oyster)	f290 Oyster extract	n.a.	f290 *Ostrea edulis* extract	n.a.

Mussels	F37 Blue mussel: *Mytilus edulis* (Canned mussel)	f37 Blue mussel extract	*Mytilus galloprovincialis* extract	f37 *Mytilus edulis* extract	n.a.
Scallops, pectens	F38 Scallop: Pecten spp. (muscle)	f338 Scalop extract		f 338 Pecten spp. extract	n.a.
Clams, cockles, arkshells	F207 Clam: Ruditapes spp.	f207 Clam extract;	*Venus gallina* extract; **Ven ga 1** from *Venus gallina*	f207 Ruditapes spp. extract	n.a.
Squids, cuttlefishes, octopuses	F59 Octopus: *Octopus vulgaris* (Fresh frozen muscle); F58 Pacific squid, Japanese flying squid: *Todarodes pacificus* (Squid meat); F258 Squid: Loligo spp. (Squid meat)	f58 Pacific squid extract; f59 Octopus extract; f258 Squid extract	Octopus vulgaris extract; *Uroteuthis duvauceli* extract; **Uro du 1** from *Uroteuthis duvauceli*	f 258 Loligo spp. extract	n.a.
Abalones	F346 Abalone: Haliotis spp.	n.a.	n.a.	n.a.	n.a.

Section S5. Cross-reactivity of TPM from economically most relevant species of shellfish with WHO registered allergens

Query: H6BD84 · H6BD84_OSTED Query ID: lcl|Query_1285587 Length: 138

>tropomyosin [Magallana gigas]
Sequence ID: ARX70262.1 Length: 284
Range 1: 127 to 260

Score:109 bits(272), Expect:1e-34,
Method:Compositional matrix adjust.,
Identities:74/134(55%), Positives:98/134(73%), Gaps:0/134(0%)

```
Query  1
RGTLESRQRTDETRMDDMESKMRQLTDVADQSENRYTEAARKLCVLEGELERAEERYELA  60
         R  LE+     E R D +E ++ +    +A++++ +Y EAARKL + E +LERAE R E A
Sbjct  127
RKVLENLNNASEERTDVLEKQLTEAKLIAEEADKKYDEAARKLAITEVDLERAEARLEAA  186

Query  61
ESKVKTLEDELHVATNSLKALEISDEKASQREDSYEETIRDLTQRLKDSQNRAEVAERRV  120
         E+KV  LE+EL V  N++K+LEIS+++ASQREDSYEETIRDLTQRLKD++NRA  AER V
Sbjct  187
EAKVLELEEELKVVGNNMKSLEISEQEASQREDSYEETIRDLTQRLKDAENRATEAERTV  246

Query  121  YTLQLENDRLSDDL  134
             LQ E DRL D+L
Sbjct  247  SKLQKEVDRLEDEL  260
```

Sequence alingment of tropomyosin seqence ID H6BD84 (*Ostrea edulis*) and tropomyosin sequence ID ARX70262.1 (*Crassostrea gigas/Magallana gigas*).
